# The impact of gadolinium doping on physicochemical properties and neuroprotective activity of polyacrylic acid conjugated cerium oxide nanoparticles – in vitro study of potential theranostics for neurodegenerative diseases

**DOI:** 10.1007/s43440-026-00863-5

**Published:** 2026-05-09

**Authors:** Rugmani Meenambal, Tomasz Kruk, Piotr Warszyński, Natalia Łopuszyńska, Władysław P. Węglarz, Katarzyna Stan-Głowińska, Joanna Wojewoda-Budka, Krzysztof Jasiński, Aleksandra Mąsior, Danuta Jantas

**Affiliations:** 1 Maj Institute of Pharmacology, Polish Academy of Sciences, Smętna 12, Kraków, 31-343 Poland; 2 Jerzy Haber Institute of Catalysis and Surface Chemistry, Polish Academy of Sciences, Niezapominajek 8, Kraków, 30-239 Poland; 3 Henryk Niewodniczański Institute of Nuclear Physics, Polish Academy of Sciences, Radzikowskiego 152, Kraków, 31-342 Poland; 4 Institute of Metallurgy and Materials Science, Polish Academy of Sciences, Reymonta 25, Kraków, 30-059 Poland; 5 Present Address: Quro Health and Lifestyle Private Limited, Thiruvananthapuram, India

**Keywords:** Gadolinium-doped nanoparticles, Theranostics, Neuroprotection, MRI, Parkinson’s disease, SH-SY5Y cells, 6-OHDA, H_2_O_2_

## Abstract

**Background:**

Cerium oxide nanoparticles (CeO NPs) are showing neuroprotective effects in various experimental models of neurodegeneration. Doping of nanoparticles with magnetic resonance imaging (MRI) contrast agents (e.g., gadolinium) could enable simultaneous diagnosis and treatment of neurodegenerative diseases, a technology called theranostics that is used primarily in oncology but can also be successfully applied in the diagnosis and treatment of neurodegenerative diseases.

**Methods:**

In this study, we doped polyacrylic acid conjugated cerium oxide nanoparticles with gadolinium (Gd-CeO) to create a theranostic agent with MRI capabilities and neuroprotective properties. These nanoparticles were evaluated for their physicochemical characteristics, magnetic resonance imaging potential, biosafety profile, cellular uptake, and neuroprotective effects compared to CeO nanoparticles (CeO) in a human neuronal model of Parkinson’s disease employing undifferentiated and retinoic acid-differentiated SH-SY5Y cells.

**Results:**

The synthesized Gd-CeO nanoparticles showed good stability, concentration-dependent T_1_ and T_2_ contrast features, and were not cytotoxic. The Gd-CeO nanoparticles were rapidly taken by cells and maintained neuroprotective potency against hydrogen peroxide (H_2_O_2_)- and 6-hydroxydopamine (6-OHDA)-induced cell damage to a similar extent as did CeO nanoparticles without Gd doping. Moreover, we demonstrated a protective effect of Gd-CeO and CeO nanoparticles on mitochondrial membrane potential, DNA fragmentation, and the number of necrotic cells in both models of cell injury, whereas at the level of caspase-3 activity, we showed an inhibitory effect of the studied NPs only in the 6-OHDA model. Finally, the protection mediated by Gd-CeO and CeO nanoparticles against H_2_O_2_ was confirmed in mouse primary cortical neurons.

**Conclusions:**

Since the developed Gd-CeO nanoparticles showed promising contrast features, as well as maintaining biosafety and neuroprotective properties similar to those of nanoparticles without Gd doping, they could be further investigated as a potential theranostic probe for neurodegenerative diseases, including Parkinson’s disease.

**Graphical Abstract:**

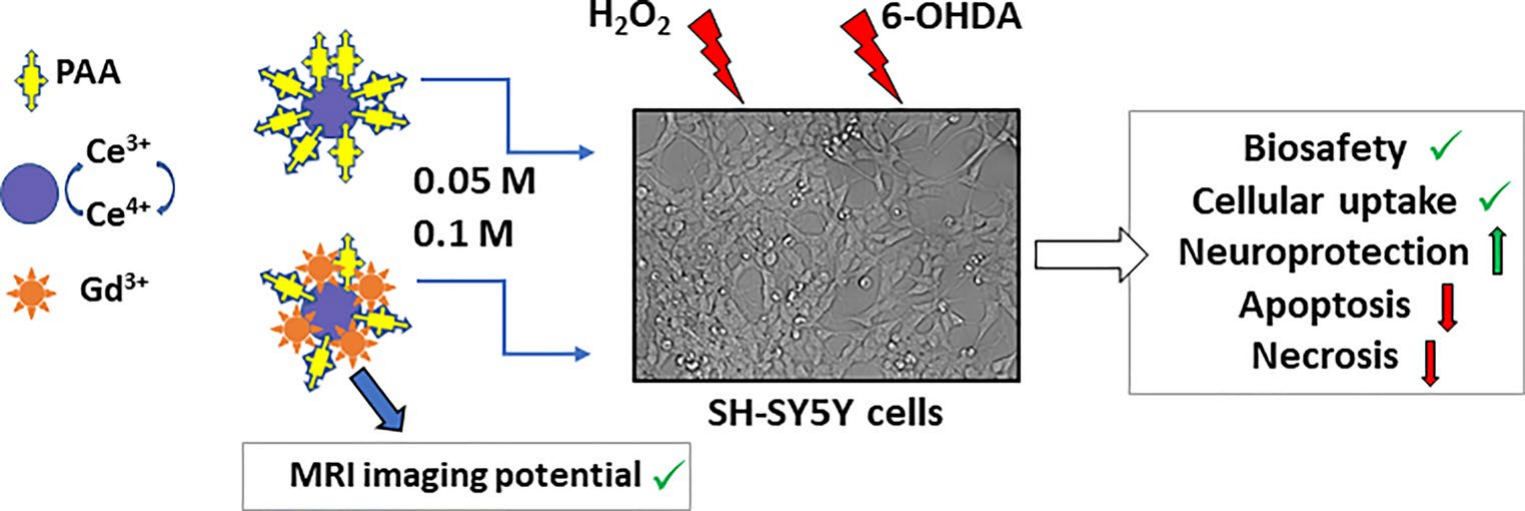

**Supplementary Information:**

The online version contains supplementary material available at 10.1007/s43440-026-00863-5.

## Introduction

Theranostic nanoparticles (NPs) play a crucial role in advancing personalized medicine by enabling the simultaneous diagnosis and treatment of diseases in a targeted manner. These NPs are designed to carry imaging agents for diagnostics and therapeutic drugs for treatment, allowing for real-time monitoring of disease progression and treatment efficacy. With the growing interest in precision medicine, theranostic NPs show significant potential for improving patient outcomes and minimizing the risk of adverse events associated with traditional systemic drug delivery [1–3]. The effectiveness of multifunctional hybrid NPs has been particularly deeply studied for many years in the cancer field, especially in targeted delivery of chemotherapy drugs, which enabled real-time imaging of tumor response to treatment and improved the survival rate of cancer patients [4–7]. It should be noted that in recent years, theranostic NPs have garnered substantial interest for their potential applications in brain delivery and treatment of neurodegenerative disorders (ND), demonstrating the ability to transport therapeutic agents across the blood-brain barrier (BBB) and into the brain [8–11]. The key requirement for using theranostic NPs in neuroprotection is their ability to cross the BBB, which poses a significant challenge in delivering drugs to the brain. These NPs can be surface-engineered to cross the BBB and actively target specific cells or regions of the brain, enhancing the efficiency and effectiveness of drug delivery. Additionally, incorporating imaging agents into the NPs allows for non-invasive monitoring of drug distribution, accumulation, and therapeutic response in real-time, providing valuable insights for optimizing treatment strategies [12–13]. Nanotheranostics can be used for early detection of biomarkers associated with ND, like amyloid plaques and neurofibrillary tangles in Alzheimer’s disease, the aggregates of alpha-synuclein protein in Parkinson’s disease (PD), or more general processes (e.g., neuroinflammation or neuronal loss) involved in neurodegeneration [10, 14–16]. Recently, cerium oxide NPs (CeO NPs) have been gaining recognition for their potential in protecting neurons, particularly concerning acute brain injuries (traumatic brain injury or ischemic stroke) or age-related chronic ND [16–19]. These NPs are distinguished by their unique ability to switch between oxidation states (Ce^3+^ and Ce^4+^), enabling them to neutralize reactive oxygen species (ROS) effectively. That antioxidant capability is crucial for CeO NPs neuroprotective action, as oxidative stress is a major contributor to neuronal damage during neurodegeneration [20–21]. In our previous research reports, we demonstrated that polyacrylic acid (PAA)-conjugated CeO NPs, as well as europium (Eu^3+^) doped CeO NPs, show neuroprotective potential in human neuroblastoma SH-SY5Y cells against cell damage induced by oxidative stress inducers, hydrogen peroxide (H_2_O_2_) and 6-hydroxydopamine (6-OHDA) [22–23].

Gadolinium (Gd) complexes are widely applied in magnetic resonance imaging (MRI), serving as highly effective and medically approved contrast agents [24]. Their paramagnetic characteristics significantly increase the relaxation rates of water protons, leading to enhanced contrast and clearer imaging of tissues, especially in the brain, which makes them advantageous for diagnosing and monitoring neurological conditions. When combined with therapeutic agents, Gd can enable simultaneous diagnosis and treatment [25]. However, there is some evidence that Gd could be deposited in the brain for months or years, and this could be particularly detrimental for children, whose brains are more vulnerable to exogenous toxins compared to adults [26]. Apart from the potential cytotoxic and genotoxic effects of Gd-based contrast agents [27], there are also concerns about their probable ecotoxicity [28]. That indicates the need to tune the optimal concentration of Gd^3+^ in nanoformulations and/or search for new alternative MRI contrast agents. The concept of tagging CeO NPs with Gd aims to merge their neuroprotective properties stemming from the antioxidant capabilities of cerium oxide with the imaging benefits of Gd, thereby enhancing their utility for both therapeutic and diagnostic applications in ND. A few studies have reported on cerium (Ce) and Gd for MRI, including research on biocompatibility of heavily Gd-doped CeO NPs as MRI contrast agents towards human mesenchymal stem cells [29], as well as the synthesis and characterization of Gd-doped CeO ceramics [30], although their neuroprotective potential remained unexplored. In a recently published paper, Nan et al. [18] demonstrated that the incorporation of Gd into Ce NPs not only was well tolerated and enabled MRI imaging of injured brain tissue, but also enhanced cerium antioxidant properties and significantly improved the brain recovery in a murine ischemic stroke model.

In this study, we developed Gd-doped PAA-conjugated CeO NPs (Gd-CeO) to create a theranostic agent with MRI capabilities and neuroprotective properties. These Gd-CeO NPs were evaluated for their physicochemical characteristics, biosafety profile, and neuroprotective effects compared to PAA-conjugated CeO NPs (CeO), using a human neuronal model of PD (undifferentiated and retinoic acid-differentiated SH-SY5Y cells) exposed to oxidative stress inducers, H_2_O_2_ and 6-OHDA [31–32]. Moreover, some mechanisms of neuroprotection mediated by synthetized NPs in SH-SY5Y cells were investigated at the level of intracellular reactive oxygen species (ROS), calpain activity, mitochondria (mitochondrial membrane potential), lysosomes (cathepsin D activity), apoptosis (caspase-3 activity and DNA fragmentation) and necrosis (propidium iodide staining) markers which are involved in pathogenesis of various ND [21, 31, 33–34]. Finally, the neuroprotective potency of CeO and Gd-CeO was analyzed in mouse primary cortical neurons exposed to oxidative stress (H_2_O_2_) or excitotoxic (glutamate) inducers.

## Materials and methods

### Materials

Cerium (III) nitrate hexahydrate (#10294-41-4), ammonium cerium (IV) nitrate (#16774-21-3), gadolinium (III) nitrate hexahydrate Gd(NO_3_)_3_.6H_2_O (#19598-90-4), polyacrylic acid sodium salt (Mw = 5100, #9003-04-7), ammonium hydroxide solution (30% NH_3_ in H_2_O, #1336-21-6), and fluorescein isothiocyanate isomer I (#F7250) were obtained from Sigma-Aldrich Chemie GmbH (Taufkirchen, Germany). For cell culture work, Dulbecco’s Modified Eagle Medium (DMEM, #41966029), trypsin/EDTA 0.25% solution (#25200056), heat-inactivated fetal bovine serum (FBS, #10500064), penicillin-streptomycin solution (#15140-122), Neurobasal A medium (#12349015), supplement B27 without antioxidants (#10889038), FluoroBrite™ DMEM (#A1896701), and Dulbecco’s phosphate-buffered saline (DPBS, without calcium and magnesium, #14190144) were purchased from Gibco (Life Technologies Ltd., Paisley, UK). The cytotoxicity detection kit (LDH release assay, #11644793001), In Situ Cell Death Detection Kit Fluorescein (#11684795910), and protease inhibitor cocktail (#11836153001) were supplied by Roche Diagnostic GmbH (Mannheim, Germany). Caspase-3 (Ac-DEVD-AMC, # ALX-260031-M001) and Cathepsin D (AMC-Gly-Lys-Pro-Ile-Leu-Phe-Phe-Arg-Leu-Lys(Dnp)-D-Arg-NH2, #BML-P145-0001) fluorogenic substrates were from Enzo Life Sciences (New York, NY, USA). CM-H_2_DCFDA assay (#C6827) and PierceTM BCA Protein Assay (#23209) were purchased from Invitrogen (Life Technologies Corporation, Eugene, OR, USA) and Thermo Scientific (Rockford, USA), respectively. Primary antibody for anti-spectrin α II (sc-48382) was purchased from Santa Cruz Biotechnology Inc. (CA, USA). A 12–230 kDa separation microplate kit (# SM-FL004), anti-Mouse Detection Module (#DM-002), and the Protein Normalization Module (#DM-PN02) were obtained from Bio-techne (Minneapolis, MN, USA). Additionally, dimethyl sulfoxide (DMSO, #D5879), Triton X-100 (#9036-19-5), N-acetyl cysteine (NAC, #A9166), propidium iodide (#P4170), stabilized hydrogen peroxide solution (30% H_2_O_2_, #H1009), 6-hydroxydopamine hydrochloride (#H4381), Ac-DEVD-CHO (#A0835), CHAPS hydrate (#C9426), DL-Dithiotreitiol (#D9779), pepstatin A (#P5318), leupeptin (#L2884), sodium citrate dihydrate (#567446), tetramethylrhodamine ethyl ester perchlorate (TMRE, #87917), retinoic acid (#R2625), RIPA Lysis Buffer 10X (#20–188), poly-L-ornithine (#), trypsin (#T4799), DNAse I (#D5025), L-Glutamine (#G8540), L-glutamic acid (glutamate, #49450) and 3-[4,5-dimethylthiazol-2-yl]-2,5-diphenyltetrazolium bromide (MTT, #M2128) were acquired from Sigma-Aldrich Chemie GmbH (Taufkirchen, Germany).

### Synthesis of Gd-CeO NPs

The CeO NPs were prepared as described in detail in our previous study [22]. Briefly, NPs designated as 0.05 M CeO and 0.1 M CeO NPs were prepared by mixing a solution of 50 mM and 100 mM cerium(III) nitrate hexahydrate salt and ammonium cerium(IV) nitrate salt with 10% by weight of PAA (MW 5100), and 2 ml of 30% ammonium hydroxide solution. Typically, the total volume of the prepared nanoparticle suspension was 20 mL. The Gd-doped CeO NPs were synthesized in a similar manner. During the synthesis of Gd-doped CeO NPs, the concentration of ammonium cerium(IV) nitrate salt - (NH_4_)_2_[Ce(NO_3_)_6_] remained constant at 50 mM and 100 mM. The concentration of cerium(III) nitrate hexahydrate salt - Ce(NO_3_)_3_·6H_2_O, was adjusted depending on the desired degree of doping with the addition of gadolinium (III) nitrate hexahydrate salt - Gd(NO_3_)_3_.6H_2_O, ensuring that the total concentration of cerium nitrate hexahydrate and gadolinium nitrate hexahydrate salts was 50 mM and 100 mM, respectively. For example, for 20% Gd doping, 0.05 M CeO, 0.01 M Gd salt, and 0.04 M Ce salt were used. The resulting suspensions of doped Gd-CeO NPs were continuously stirred for 24 h and dialyzed against 5 L of distilled water at pH 7 for 2 days, with the water being changed three times a day. Different Gd contents in the CeO nanoparticle matrix (1–30%) were experimentally optimized (data not shown). For the biological studies, the highest achievable content was selected to obtain the strongest possible imaging signal while maintaining the stability of the nanoparticle suspension. This approach was analogous to that used in the previous work on Eu-CeO NPs [23]. As a result, the suspensions used for further studies were 0.05 M CeO, 0.05 M 20% Gd-CeO, 0.1 M CeO, and 0.1 M 20% Gd-CeO, and they were stored in the dark at room temperature.

### Characterization of CeO and Gd-CeO NPs

The zeta potential and hydrodynamic particle size of all the synthesized samples (0.05 M CeO, 0.05 M 20% Gd-CeO, 0.1 M CeO, and 0.1 M 20% Gd-CeO) were determined by Dynamic Light Scattering (DLS) (Zetasizer Ultra Red Series, Malvern Instruments, United Kingdom). It was done at 25 °C in triplicate with at least 20 measurements using water as dispersant with parameters set for cerium oxide (refractive index = 2.2 and absorption = 0.001). Zeta potential measurements were conducted using Laser Doppler Electrophoresis and also by Zetasizer Ultra Red Series. Nanoparticle concentration was measured by Dynamic Light Scattering (DLS) with Multi-Angle Dynamic Light Scattering (MADLS^®^). The instrument recorded the time-averaged photon count rate of the scattered light at multiple angles (173°, 90°, and 13°). Samples were appropriately diluted to avoid multiple scattering and filtered (0.2 μm). Measurements were performed at a controlled temperature (typically 25 °C) in disposable cuvettes. Results were reported as particle number concentration (particles/mL), with measurements typically performed in triplicate to ensure reproducibility. The evaluated error of the concentration determination was 10%.

Scanning electron microscope images for 0.1 M 20% Gd-CeO NPs were determined by JEOL JSM-7500 F model, Jeol, Tokyo, Japan. High-resolution imaging and chemical analysis of the NPs were also performed using a probe-corrected THEMIS transmission electron microscope (TEM) operated at 200 kV and equipped with an X-FEG source, a HAADF-STEM detector, and a Super-X EDX system. For TEM analysis, a drop of nanoparticle suspension was deposited onto a carbon-coated copper grid and dried in air.

### Stability testing of suspensions of CeO and Gd-CeO NPs

The NPs size, polydispersity index (PDI), and zeta potential were monitored over time to determine the changes in formed NPs according to the protocols described above. The tested NPs were stored in a suspension in the dark at room temperature, and their stability was regularly determined after 1, 2, 3, 6, 9, 12, 15, and 20 months of storage.

### Magnetic resonance relaxometry and imaging

Four sets of eight consecutive dilutions with decreasing concentrations were prepared from the initial suspensions of 0.05 M (S1) and 0.1 M (S3) of CeO and 0.05 M (S2) and 0.1 M (S4) Gd-CeO (with 20% Gd substitution), to investigate MRI contrasting properties and perform T_1_ and T_2_ relaxation measurements. The resulting NPs concentrations in the series of dilutions were: 50; 25; 12.5; 6.25; 3.125; 1.5625; 0.78125; 0.390625 mM for samples S1 and S3, while 100; 50; 25; 12.5; 6.25; 3.125; 1.5625; 0.78125 mM for samples S2 and S4, respectively. The corresponding concentrations of Gd (for samples S3 and S4) were 20% of the NPs concentrations.

The T_1_ and T_2_ relaxation measurements and imaging were performed on a 9.4T preclinical MRI scanner, Bruker Biospec 94/20. The RARE VTR sequence was used to determine the values of T_1_ relaxation times for individual samples. The imaging geometry parameters were as follows: layer thickness: 1 mm, matrix 96 × 96, field of view 4.5 × 4.5 cm. The echo time value (TE) was set to 6.0 ms. The number of T_1_ experiments was 13, and the effective repetition time TR values were: 11, 31, 71, 91, 191, 391, 791, 1491, 1991, 2491, 4991, 9991, 14,991 ms. The MSME sequence with the following parameters was used to measure the T_2_ relaxation time: layer thickness: 1 mm, MTX: 96 × 96, FOV: 4.5 × 4.5 cm, TR: 15,000 ms. To sample the transverse magnetization decay curve, 256 spin echoes repeated every 6.5 ms were used, covering the time between 6.5 and 1664 ms. For the obtained relaxation times, straight lines were fitted to determine the molar relaxivity r_1_, r_2_ according to the formula:

$$\:{R}_{\mathrm{1,2}}={R}_{S1,\:S2}+{r}_{1,\:2}\cdot\:c$$

where $\:{R}_{\mathrm{1,2}}\:=\:\frac{1}{{T}_{\mathrm{1,2}}}$, and $\:{R}_{S1,\:S2}$ – are relaxation rate of the medium (i.e. water) without NPs, respectively, *\:c* – is gadolinium or nanocarriers concentration in the sample, $\:{r}_{1,\:2}$ – is molar relaxivity.

### SH-SY5Y cell culture

Human neuroblastoma SH-SY5Y cells were obtained from the American Type Culture Collection (CRL-2266, ATCC, Manassas, VA, USA). The cells were cultured in DMEM supplemented with 10% (v/v) FBS and 1% (v/v) penicillin/streptomycin and maintained at 37 °C in a humidified atmosphere containing 95% air and 5% CO_2_, as described previously [22]. The cells after trypsinization and manual counting (Bürker chamber) were seeded for experiments with undifferentiated SH-SY5Y (UN-SH-SY5Y) cells at densities of 4 × 10^4^, 2 × 10^5^, and 1 × 10^6^ cells per well in 96-, 24-, and 6-well plates, respectively. For experiments with differentiated SH-SY5Y cells (RA-SH-SY5Y), the cells were seeded at densities 2 × 10^4^, 1 × 10^5^, and 5 × 10^5^ cells per well in 96-, 24-, and 6-well plates, respectively, and cultured in medium containing 10 µM retinoic acid (RA) for 6 days (with medium changes every 2 days). One day before treatment, the culture medium in UN- and RA-SH-SY5Y cells was replaced with experimental medium containing 1% (v/v) FBS. For experiments, the cells were used between passages 5–15. To confirm the absence of Mycoplasma contamination, cells were regularly tested using the MycoBlue™ Mycoplasma Detector (#D101-01, Vazyme Biotech Co., Ltd., Nanjing, China).

### Cell treatment

Initially, both UN- and RA-SH-SY5Y cells were treated with varying dilutions (10x and 20x) of 0.05 M CeO, 0.05 M 20% Gd-CeO, 0.1 M CeO, and 0.1 M 20% Gd-CeO NPs for 24 and 48 h to evaluate NPs biosafety. Subsequently, to investigate the protective potential of the NPs, cells were pre-treated with NPs (dilutions 10x, 20x and 40x) or vehicle (10% v/v distilled water) for 30 min, followed by a 24-hour exposure to H_2_O_2_ (IC_50_ concentrations of 0.375 and 0.5 mM for UN-SH-SY5Y and RA-SH-SY5Y cells, respectively) and 6-OHDA (IC_50_ concentrations of 0.1 mM and 0.2 mM for UN-SH-SY5Y and RA-SH-SY5Y cells, respectively). The chosen concentrations of H_2_O_2_ and 6-OHDA were optimized in our previous study, and as a positive control in both oxidative stress models, an antioxidant, N-acetyl cysteine (NAC, 1mM) was employed [22]. For verification of assays for caspase-3 or Cathepsin D activity, we used relevant inhibitors of these enzymes, Ac-DEVD-CHO (20 µM) or pepstatin A (0.3 µM).

Stock solutions (100x) of H_2_O_2_ and 6-OHDA were made in distilled water on the experiment day. The NAC (100 mM) stock solution was prepared in distilled water, whereas the stock solutions of Ac-DEVD-CHO (2 mM) and pepstatin A (30 µM) were prepared in DMSO, and stored between independent experiments at -20 °C. The Glu (100 mM) stock solution was prepared in 100 mM NaOH immediately before use. The cell-damaging agents and positive controls were added to cells at a volume of 1% (v/v). The synthesized NPs were stored in solutions at room temperature in a dark place. For all experimental groups without NPs, the vehicle (10% v/v distilled water) was added. All experiments were done under limited light exposure conditions.

### FITC labelling of CeO and Gd-CeO NPs and their cellular uptake

To label CeO and 20% Gd-CeO NPs with fluorescence, we used a procedure described in our previous work [22]. The absorption spectra of CeO, FITC (fluorescein isothiocyanate), and FITC-labeled 20% Gd-CeO NPs were recorded using a UV-Vis Spectrometer (Shimadzu Corporation) across a wavelength range of 200 to 800 nm. To assess the cellular uptake of NPs, cells were seeded in 24-well plates and treated with FITC-conjugated NPs (10% v/v) at concentrations of 0.05 M CeO, 0.05 M 20% Gd-CeO, 0.1 M CeO, and 0.1 M 20% Gd-CeO NPs for 0.5 h, 1 h, 2 h, 3 h, and 6 h in both UN- and RA-SH-SY5Y cells. The control cells received vehicle (10% v/v distilled water) treatment. Following treatment, the cells were washed and first observed using an inverted fluorescence microscope (AxioObserver, Carl Zeiss, Jena, Germany) and next collected on ice and analyzed with the BD FACS Canto II System and BD FACSDiva™ v5.0.1 Software (BD Biosciences, San Jose, CA, USA) at FITC (green fluorescence) channel as described in details previously [22]. The mean fluorescence intensity and the percentage of FITC-positive cells were measured for each sample. The data are presented as the FITC mean fluorescence intensity (± SEM) from two independent experiments, each conducted in duplicate.

### Cytotoxicity assay

For the assessment of cell damage in SH-SY5Y and primary cortical neurons, the Cytotoxicity Detection Kit (Roche, #11644793001) was used, which is based on the measurement of the released from cells lactate dehydrogenase (LDH) into culture medium after particular treatments, as described previously [22]. Absorbance for each sample was measured at 490 nm with a multi-well plate reader (Infinite^®^ M200 PRO, Tecan Austria GmbH, Grodig, Austria). The data after normalization to the vehicle (10% v/v distilled water)-treated cells are expressed as the mean ± SEM from 2 to 9 independent experiments, each with 3–5 replicates.

### Microscopic assessment of morphological changes

To confirm the neuroprotective effects of 0.05 M CeO, 0.05 M 20% Gd-CeO, 0.1 M CeO, and 0.1 M 20% Gd-CeO NPs against H_2_O_2_ and 6-OHDA in both UN-SH-SY5Y and RA-SH-SY5Y cells, found in the LDH test, the cells growing in a 24-well plate format after 24 h of treatment were transferred to FluoroBrite™ DMEM and imaged using an inverted fluorescence microscope (AxioObserver, Carl Zeiss, Jena, Germany). The differential interference contrast (DIC) technique was employed for the microscopic evaluation, and images were captured using a black-and-white camera (Axio-CamMRm, Carl Zeiss, Jena, Germany).

### Propidium iodide staining and flow cytometry

To evaluate the impact of tested NPs on necrotic markers, UN- and RA-SH-SY5Y cells cultured and treated as described in the section above (DIC imaging) were stained with propidium iodide (PI) as described previously [22]. Approximately 1 × 10^4^ cells were analyzed by the BD FACS Canto II System and BD FACSDiva™ v5.0.1 Software (BD Biosciences, San Jose, CA, USA) at the PerCP-Cy5-5-A fluorescence channel (red fluorescence). The PI-positive cells are reported as a percentage of the total damaged cell population (± SEM) from 2 to 3 independent experiments, each performed in duplicate.

### Caspase-3 activity assay

Since our previous studies demonstrated the involvement of caspase-3 inhibition, an apoptotic marker, in protection mediated by CeO NPs against 6-OHDA- but not H_2_O_2_-evoked cell damage in UN- and RA-SH-SY5Y cells [22–23], we measured the activity of this cysteine protease enzyme after treatment of both cell phenotypes with CeO and 20% Gd-CeO NPs and 6-OHDA. The cells after treatment (pretreatment with vehicle (10% v/v distilled water), NPs or caspase-3 inhibitor, Ac-DEVD-CHO (20 µM) for 30 min followed by 18 h exposure to 6-OHDA (0.1 and 0.2 mM for UN- and RA-SH-SY5Y cells, respectively)) were frozen and kept at -20 °C. The measurement of caspase-3 activity and data analysis were performed as described in detail in our previous work [22]. The data after normalization to protein content were expressed as a percentage of vehicle (10% v/v distilled water)-treated cells and are presented as the mean ± SEM from 3 independent experiments, each with duplicates.

### TUNEL labelling and flow cytometric detection of apoptotic cells

To identify apoptotic fragments of DNA, the TUNEL (terminal deoxynucleotidyl transferase (TdT)-mediated dUTP nick-end labeling) assay was performed according to the manufacturer’s instructions (In Situ Cell Death Detection Kit Fluorescein, Roche Diagnostic) as described previously [35]. The RA-SH-SY5Y cells growing in a 6-well plate format after treatment were collected on ice, centrifuged (1300 rpm, 5 min. RT), washed with ice-cold DPBS, fixed in 4% paraformaldehyde for 20 min at room temperature, and again washed with DPBS twice. Next, the cells were digested (0.1% solution of sodium citrate and 0.1% Triton-X_100_ in PBS) for 2 min on ice and stained for 60 min at 37 °C with the TUNEL solution. After washing with DPBS, approximately 1 × 10^4^ cells were analyzed using the BD FACS Canto II System and BD FACSDiva™ v5.0.1 Software (BD Biosciences, San Jose, CA, USA) in the fluorescence channel for FITC (green fluorescence). The TUNEL-negative cells were considered to be undamaged, and the TUNEL-positive cells were considered to be apoptotic. Data are presented as a percentage of TUNEL-positive cells (± SEM) established from 3 independent experiments, each with duplicates.

### Measurement of the intracellular ROS level

Intracellular ROS level was measured in RA-SH-SY5Y cells with CM-H_2_DCFDA probe as described previously [22–23]. Briefly, the cells cultured in 96-well plate format after differentiation for seven days with RA were loaded with 5 µM CM-H_2_DCFDA (in FluoroBrite™ DMEM) and after 5 min, treated with 0.05 M CeO or 0.05 M 20% Gd-CeO NPs for 25 min, and exposed for the next 30 min to 1 mM H_2_O_2_. The antioxidant NAC (1 mM) was used as a positive control for the assay. Next, the cells were washed twice with pre-warmed FluoroBrite™ DMEM and fluorescence intensity was measured in a multi-well microplate reader (CLARIOstar Plus, BMG LABTECH) with excitation and emission wavelengths of 488 nm and 535 nm, respectively. Data were normalized to vehicle (10% v/v distilled water)-treated cells and are presented as the mean ± SEM from 3 independent experiments, each with 3–5 replicates.

### Measurement of Mitochondrial Membrane Potential (MMP)

MMP was measured with the fluorescent probe TMRE as described previously [36]. The RA-SH-SY5Y cells growing in a 96-well plate after treatment were washed with pre-warmed FluoroBrite™ DMEM, incubated with 100 nM TMRE for 20 min, and washed again twice. The fluorescence was measured in a multi-well microplate reader (Infinite^®^ M200 PRO, Tecan Austria GmbH, Grodig, Austria) with excitation and emission wavelengths of 540 nm and 595 nm, respectively. Data were normalized to vehicle (10% v/v distilled water) -treated cells and are presented as the mean ± SEM from 3 independent experiments, each with 3–5 replicates.

### Cathepsin D activity measurement

Since our previous studies demonstrated the involvement of cathepsin D (Cth D) activation in the mechanism of H_2_O_2_ cytotoxicity in SH-SY5Y cells [32, 37], we employed this assay in the present study to investigate possible mechanisms of neuroprotection mediated by CeO or 20% Gd-CeO NPs. The UN- and RA-SH-SY5Y cells grown in a 6-well plate after treatment were frozen and stored at -20 °C. The Cth D activity was measured in cell lysates using a fluorogenic substrate AMC-Gly-Lys-Pro-Ile-Leu- Phe-Phe-Arg-Leu-Lys(Dnp)-D-Arg-NH2 as described previously [37]. The data after normalization to protein content measured by the BCA protein assay were expressed as a percentage of vehicle (10% v/v distilled water)-treated cells and shown as the mean ± SEM from 3 independent experiments, each with duplicates.

### Western blot with ProteinSimple Jess

For Western blot analysis of spectrin α II total and cleavage products in whole cell lysates, first RA-SH-SY5Y cells cultured and differentiated in 6-well plates were pre-treated for 30 min with vehicle (10% v/v distilled water), 0.05 M CeO or 0.05 M 20% Gd-CeO at dilution 10x, followed by 18 h treatment with H_2_O_2_ (0.5 mM) or 6-OHDA (0.2 mM). After treatment, the cells were washed twice with ice-cold DPBS and lysed (75 µL/well) with RIPA buffer supplemented with protease inhibitor cocktail (Roche). The protein level was measured with BCA Protein Assay and Western blot as performed on an automated capillary-based western blot platform, ProteinSimple Jess, according to the manufacturer’s instructions (Bio-Techne). An equal amount of protein (1 µg/µL) was loaded on a 12–230 kDa separation microplate plates and a primary mouse monoclonal antibody (spectrin α II) was used in a dilution 1:50. A chemiluminescence signal was detected using the Anti-Mouse Detection Module, whereas total protein was measured with the Protein Normalization Module. Densitometric analysis of bands of interest was performed using the manufacturer-provided Compass software and normalized by the system to measure total protein levels. The data are expressed as the mean corrected area ± SEM from one experiment in duplicates.

### Primary cortical neuronal cell culture, treatment, and cell viability assessment

For verification of neuroprotection mediated by tested NPs in a more physiologically relevant neuronal in vitro system, we used mouse primary cortical neurons. Cell cultures were prepared from mouse CD1 embryos (15/16 days of gestation) taken from pregnant CD1 mice (Charles River Laboratories, Sulzfeld, Germany), and were cultured as described previously [32]. The animal care and protocol for generating the primary neuronal cell cultures were in accordance with European Union (Directive 2010/63/EU, amended by Regulation (EU) 2019.1010) guidelines on the ethical use of animals, and according to national regulations, it does not require the approval of the local ethics committee for animal research. All experiments were conducted according to the principles of the Three Rs, and all efforts were made to minimize the number of animals used and their suffering. The neuronal cells were seeded at a density of 7.5 × 10^4^ cells per well in poly-L-ornithine (0.05 mg/mL) precoated 96-well plate format and were cultured for 7 days in Neurobasal A medium supplemented with B27 (without antioxidants) and antibiotics (0.06 µg/mL penicillin and 0.1 µg/mL streptomycin) at 37 °C in a humidified atmosphere containing 5% CO_2_ with medium exchange every 2 days. At 8th DIV, primary neuronal cell cultures were first treated for 24 h with 0.1 M CeO and 0.1 M 20% Gd-CeO NPs at a 10x dilution for assessment of NPs biosafety towards this cellular system. For neuroprotection evaluation, neurons were pre-treated for 30 min. with 0.1 M CeO and 0.1 M 20% Gd-CeO NPs (dilutions 10x and 20x) followed by 24 h exposure to oxidative stress (H_2_O_2_, 0.125 mM) and excitotoxic (glutamate, Glu, 1mM) inducers. Cytotoxicity was estimated by measuring LDH released into culture medium (details in section *Cytotoxicity assay*), whereas cell viability was quantified with MTT reduction assay as described previously [32]. Absorbance of each sample for the MTT assay was measured at 570 nm with a multi-well plate reader (Infinite^®^ M200 PRO, Tecan Austria GmbH, Grodig, Austria). The data were normalized to the vehicle (10% v/v distilled water)-treated cells and expressed as a percentage of the control ± SEM established from 2 independent experiments with 3–5 replicates.

### Statistical analysis

The data were analyzed with Statistica 13.3 software (StatSoft Inc., Tulsa, OK, USA). Data normality was assessed using the Shapiro–Wilk test, and homogeneity of variance was verified using the Levene test. One- or two-way analysis of variance (ANOVA) with post hoc Duncan’s test for multiple comparisons with assumed *p* < 0.05 were used.

## Results

### Physicochemical characteristics of synthesized nanoparticles

**Fig. 1:**
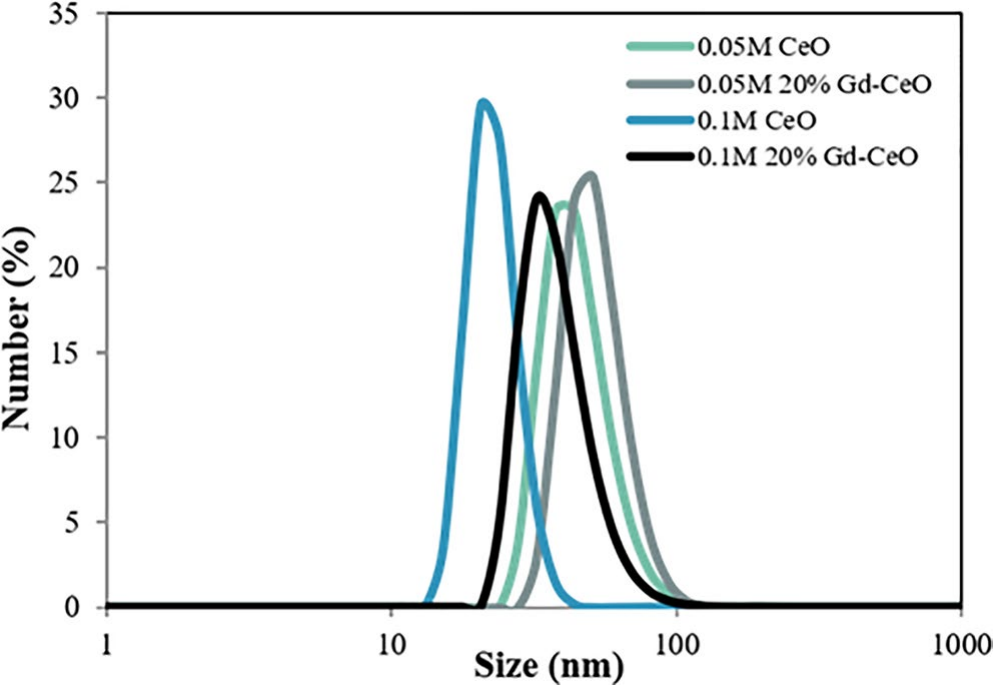
The size distributions for 0.05 M and 0.1 M CeO and 20% Gd-CeO NPs obtained by the DLS method. CeO –polyacrylic acid-conjugated cerium oxide nanoparticles; DLS – Dynamic Light Scattering; Gd-CeO – polyacrylic acid-conjugated cerium oxide nanoparticles doped with gadolinium

The size distributions for 0.05 M and 0.1 M CeO NPs, as well as 20% Gd-CeO NPs of respective concentrations, were determined using the DLS method. They are illustrated in Fig. 1. The analysis revealed that the average size of the CeO 0.05 M NPs ranged from 40 to 50 nm, while the 0.1 M NPs exhibited a smaller size range of 20 to 30 nm. Gd^3+^ doping in the Ce matrix caused a slight increase in size (distribution by number) of ca. 10 nm, both for 0.05 M and 0.1 M 20% Gd-CeO NPs.

The zeta potential of NPs, being the measure of their surface charge, is a key parameter for assessing the electrostatic stability of a colloidal system. A high absolute value of zeta potential, typically greater than +/-30 mV, indicates strong electrostatic repulsion between particles. This repulsion prevents aggregation (flocculation or coalescence), thereby indicating a stable system. Conversely, values closer to zero suggest weaker repulsive forces, making the system prone to instability. For the synthesized CeO and 20% Gd-CeO NPs, the measured zeta potential values consistently exceeded +/-30 mV for all samples (exactly between − 35 and − 45 mV, cf. Table 1), confirming the electrostatic stability of the obtained in the aqueous suspension. The polydispersity index (PDI) is a crucial indicator of the homogeneity of a nanoparticle size distribution. A low PDI value indicates a more monodispersed system, meaning the particles are largely uniform in size. Generally, a PDI below 0.2 indicates good monodispersity, while values exceeding 0.3 suggest a polydisperse system with a wide range of particle sizes. A high PDI can also hint at aggregation, indicating potential instability. For the synthesized CeO and 20% Gd-CeO NPs, the PDI values were between 0.2 and 0.25, indicating relative monodispersity. The size and zeta potential of CeO and 20% Gd-CeO NPs were monitored for nearly 2 years to investigate the long-term stability of the system. The suspensions of NPs were stored at room temperature in the dark. The measurements were conducted at 1, 2, 3, 6, 9, 12, 15, and 20 months after preparation as demonstrated in Fig. S1. Neither, the values PDI nor the the zeta potential changed over the measured period beyond the statistical error range (data not shown). A more accurate representation of the minimal (practically negligible) changes in particle stability is provided in the accompanying figure showing particle size measurements over time (Fig. S1). Thus, we could conclude that the developed NPs were stable in time for up to 20 months.

**Table 1:** The zeta potential of 0.05 M and 0.1 M CeO and 20% Gd-CeO NPs

Suspension of NPs	Zeta potential [mV]
0.05 M CeO	**− 4**3
0.05 M 20% Gd-CeO	**− 3**7
0.1 M CeO	**− 4**0
0.1 M 20% Gd-CeO	**− 3**6

CeO – polyacrylic acid-conjugated cerium oxide nanoparticles; Gd-CeO – polyacrylic acid-conjugated cerium oxide nanoparticles; NPs – nanoparticles

Additionally, the concentration of the obtained NPs, expressed as the number of particles per milliliter determined using the DLS method with MADLS is presented in Table 2. Nanoparticle concentration expressed as their number per unit volume is then used in the analysis of cytotoxic data. In Table 2, the number of NPs in experimental plates is presented, taking into account the volume of medium in wells (100 µl) as well as different dilution factors of particular NPs (10x, 20x, 40x).

**Table 2:** NPs concentration (particles/ml) measured by DLS with MADLS and their concentrations in experimental wells under dilutions 10x, 20x and 40x

Suspension of nanoparticles	Concentration [particles/ml]	Dilution 10x [particles/well]	Dilution 20x [particles/well]	Dilution 40x [particles/well]
0.05 M CeO	1.4 × 10^12^	1.4 × 10^10^	7 × 10^9^	3.5 × 10^9^
0.05 M 20% Gd-CeO	1.9 × 10^12^	1.9 × 10^10^	9.5 × 10^9^	4.75 × 10^9^
0.1 M CeO	3.8 × 10^13^	3.8 × 10^11^	1.9 × 10^11^	9.5 × 10^10^
0.1 M 20% Gd-CeO	3.3 × 10^13^	3.3 × 10^11^	1.65 × 10^11^	8.25 × 10^10^

CeO – polyacrylic acid-conjugated cerium oxide nanoparticles; DLS – Dynamic Light Scattering; Gd-CeO – polyacrylic acid-conjugated cerium oxide nanoparticles with gadolinium; MADLS – Multi-Angle Dynamic Light Scattering; NPs – nanoparticles

**Fig. 2:**
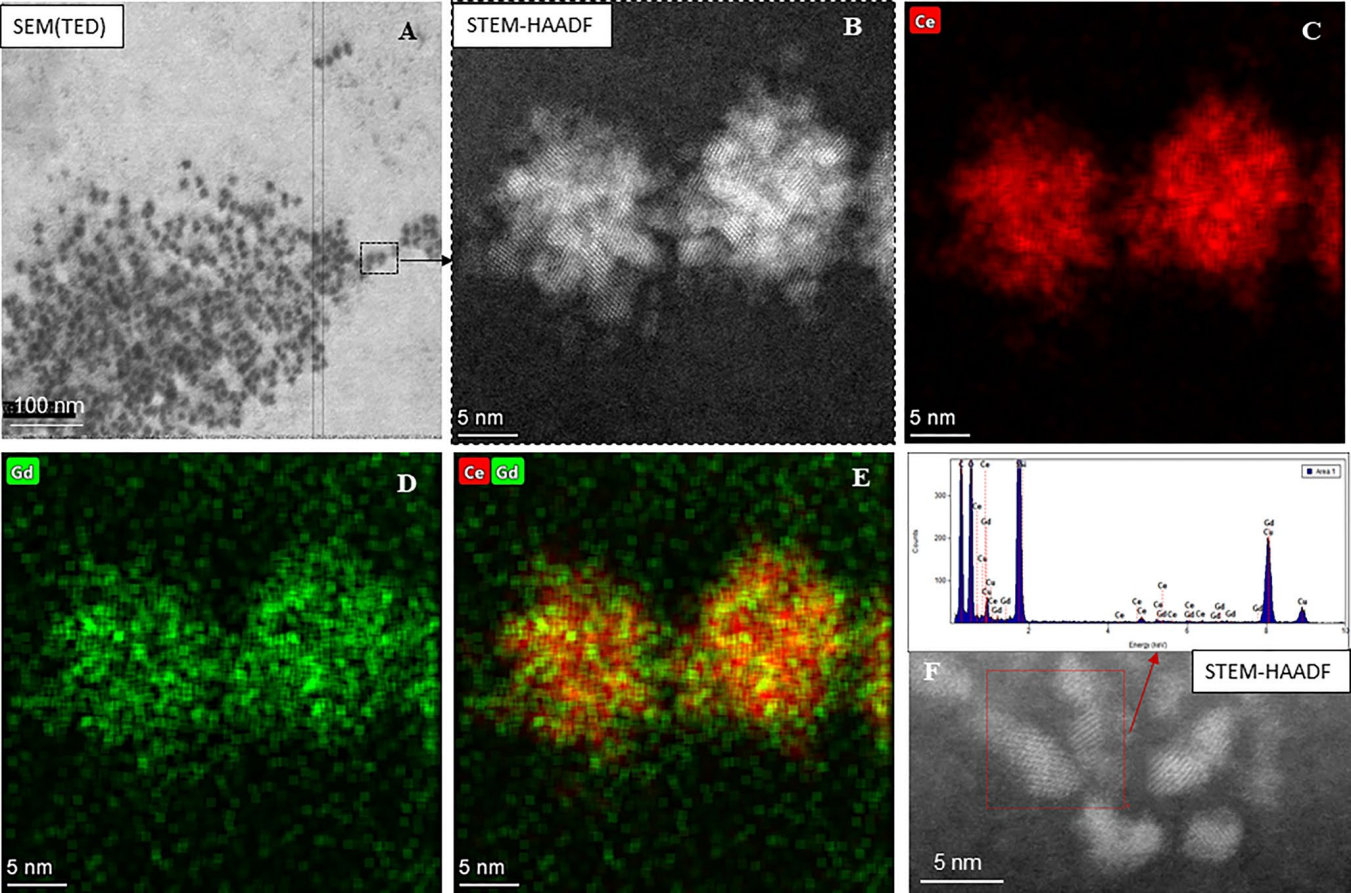
0.1 M 20% Gd-CeO NPs: (**A**) - the scanning electron microscopy image; (**B**) – the TEM image analyzed region; (**C**) – Ce map highlighted in red; (**D**) – Gd map highlighted in green; (**E**) – a superimposed image of Ce and Gd maps; (**F**) – corresponding EDS. Ce – cerium; CeO – polyacrylic acid-conjugated cerium oxide nanoparticles; EDS – energy dispersive spectrum; Gd – gadolinium; Gd-CeO – polyacrylic acid-conjugated cerium oxide nanoparticles with gadolinium; NPs – nanoparticles; TEM – transmission electron microscopy

The SEM images of 0.1 M 20% Gd-CeO NPs shown in Fig. 2A revealed a size range of ∼10–40 nm, consistent with DLS results. The elemental distribution maps of Gd in the Ce nanoparticle matrix obtained by the TEM technique are also presented, Fig. 2B shows the analyzed region of 20% Gd-CeO NPs; Fig. 2C presents the Ce elemental map highlighted in red; Fig. 2D shows the Gd map highlighted in green; and Fig. 2E presents a superimposed image of Ce and Gd maps illustrating the spatial distribution of cerium and gadolinium within NPs. The imaging results indicate that Gd is dispersed in Ce nanoparticle and appears to be well mixed within the matrix. As expected, Ce is present in a significantly higher abundance, consistent with the 20% gadolinium doping level. They indicate that Ce and Gd are intermixed, with no visible separation. The NPs exhibited a spherical morphology. Energy Dispersive X-ray (EDX) analysis at specific points on the TEM images provided elemental composition data, confirming the presence of Gd and Ce with their respective percentages, thereby verifying the successful synthesis of the Gd-CeO NPs (Fig. 2F).

### Magnetic resonance relaxometry and imaging

**Fig. 3:**
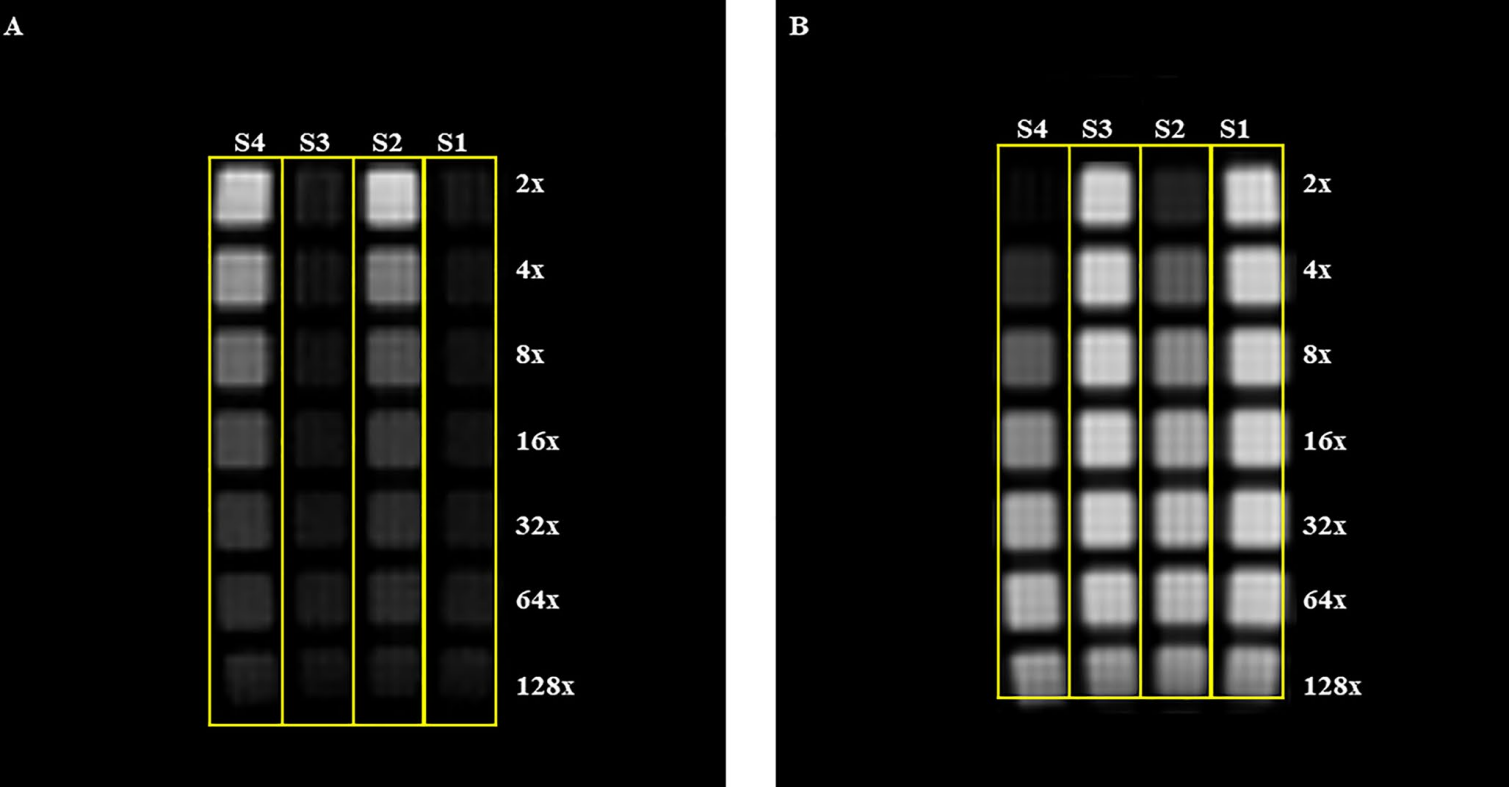
Examples of T_1_ (**A**) and T_2_ (**B**) weighted images of consecutive dilutions (up to 128x) of 20% Gd-CeO NPs and CeO NPs. Pulse sequences and parameters were as follows: RAREVTR with TR/TE equal to 40/6 ms for the T_1_-weighted image, and MSME with TR/TE equal to 15,000/45,5 ms for the T_2_-weighted image, respectively. S1–0.05 M CeO; S2–0.05 M 20% Gd-CeO; S3–0.1 M CeO; S4–0.1 M 20% Gd-CeO. CeO – polyacrylic acid-conjugated cerium oxide nanoparticles; Gd-CeO – polyacrylic acid-conjugated cerium oxide nanoparticles; NPs – nanoparticles

**Fig. 4:**
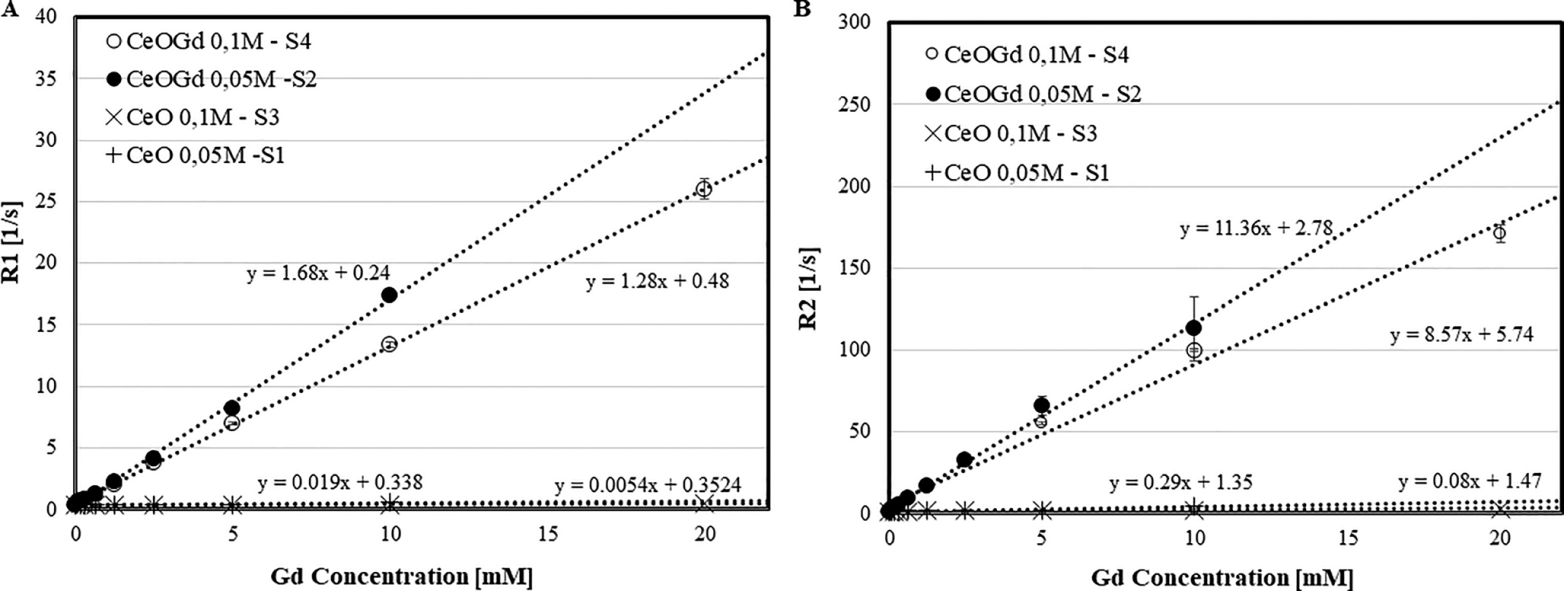
The dependence of the relaxation rate R_1_ (**A**) and R_2_ (**B**) on the concentration of Gd for 20% Gd-CeO NPs. R_1_ and R_2_ dependencies for corresponding concentrations of non-doped CeO NPs added for comparison. CeO – polyacrylic acid-conjugated cerium oxide nanoparticles; Gd – gadolinium; Gd-CeO – polyacrylic acid-conjugated cerium oxide nanoparticles; NPs – nanoparticles

From the MRI results shown in Fig. 3A, it can be concluded that CeO NPs labelled with Gd (20% Gd-CeO NPs) exhibit reasonable positive (i.e., T_1_-based) contrasting properties. For a broad range of dilution factors, there is a significant contrast between CeO and Gd-CeO NPs solutions. Negative (i.e., T_2_-based) contrast is also detectable (Fig. 3B), but only for dilutions up to 4–8 times from the original samples, which have nominal concentrations of 0.05 M – 0.1 M NPs, respectively. Detailed calculations, based on the dependence of relaxation rates R_1_ and R_2_ on Gd concentration (Fig. 4), give values of the r_1_ molar relaxivity equal to 1.7 and 1.3 mM^−1^s^− 1^ for samples S4 and S2, respectively. With a sufficient accumulation, it should be possible to detect positive contrast enhancement produced by these NPs in vivo in T_1_-weighted images. Results of the r_2_ molar relaxivity of 11.4 and 8.6 mM^−1^s^− 1^ (for S2 and S4 samples, respectively), which is relatively low compared to typical negative contrast agents, suggest that the contrast would be detectable on T_2_-weighted images only in the range of the highest concentrations. Moreover, it can be seen that the contrasting properties arise exclusively from the presence of Gd. The CeO NPs (samples S1 and S3) shorten the T_1_ and T_2_ relaxation times of water solvent only minimally, and their contrasting effect is negligible. Therefore, at concentrations higher than the detection limit, MRI could be applied for the visualisation of the distribution of CeO NPs labelled with Gd due to the contrast enhancement visible in T_1_- and to some extent also in T_2_-weighted images, as is visualised in Fig. 3.

### Biosafety evaluation of CeO and Gd-CeO NPs

A two-way ANOVA analysis of LDH release assay results showed that 24–48 h of treatment with 0.05 M and 0.1 M CeO and 20% Gd-CeO NPs at dilution 10x did not evoke any detrimental effects on UN- (treatment effect F_4,25_ = 1.7359, *p* = 0.174; time effect F_1,25_ = 0.3, *p* = 0.593; interaction treatment x time F_4,25_ = 2.7, *p* = 0.0530) or RA-SH-SY5Y cells (treatment effect F_4,14_ = 2.4, *p* = 0.098; time effect F_1,14_ = 0.2, *p* = 0.6449; interaction treatment x time F_4,14_ = 0.2, *p* = 0.941) (Table 3).

**Table 3:** Biosafety of 0.05 M and 0.1 M CeO and 20% Gd-CeO NPs in UN- and RA-SH-SY5Y cells

Sample	UN-SH-SY5Y		RA-SH-SY5Y	
	24 h	48 h	24 h	48 h
C + V	100 ± 0.00	100 ± 0.00	100 ± 0.00	100 ± 0.00
0.05 M CeO	111.85 ± 7.38	92.92 ± 3.24	109.86 ± 3.70	105.83 ± 13.10
0.05 M 20% Gd-CeO	102.61 ± 6.67	87.53 ± 1.04	113.31 ± 2.70	106.23 ± 14.87
0.1 M CeO	103.01 ± 10.61	112.11 ± 7.08	112.09 ± 4.41	111.68 ± 0.96
0.1 M 20% Gd-CeO	102.43 ± 3.47	116.65 ± 7.01	117.58 ± 4.70	120.06 ± 7.62
n	4	3	2–3	2

The UN- and RA-SH-SY5Y cells were treated with vehicle (C + V; 10% v/v distilled water), 0.05 M and 0.1 M CeO, and 20% Gd-CeO NPs at dilutions 10x for 24 and 48 h. Cytotoxicity was estimated by measurement of LDH level released into the cell culture medium after cell treatment with NPs or vehicle. The data were normalized to vehicle-treated cells (C + V) and are expressed as the mean ± SEM from 2–4 independent experiments; two-way ANOVA followed by Duncan post hoc. C – control; CeO – polyacrylic acid-conjugated cerium oxide nanoparticles; Gd-CeO – polyacrylic acid-conjugated cerium oxide nanoparticles; n – number of independent experiments; NPs – nanoparticles; V- vehicle

### NPs labelling with FITC and measurement of their cellular uptake in UN- and RA-SH-SY5Y cells

**Fig. 5:**
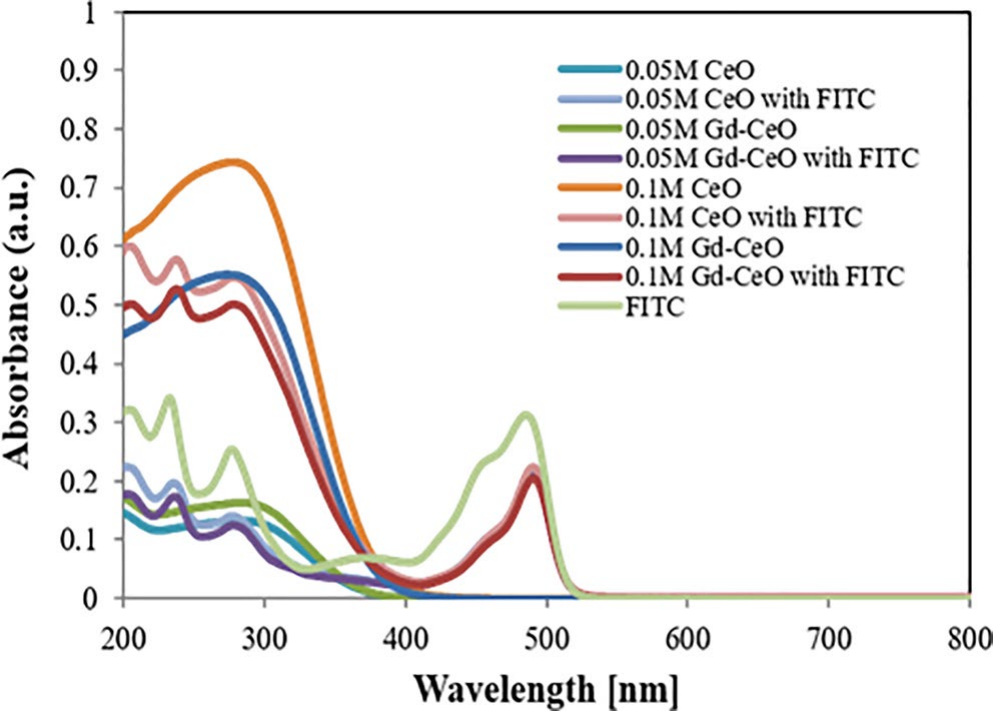
UV-VIS absorption spectra of pure FITC, 0.05 M and 0.1 M CeO, and 20% Gd-CeO NPs, and FITC labelled 0.05 M and 0.1 M CeO and 20% Gd-CeO NPs. CeO – polyacrylic acid-conjugated cerium oxide nanoparticles; FITC – fluorescein isothiocyanate; Gd-CeO – polyacrylic acid-conjugated cerium oxide nanoparticles; NPs – nanoparticles

Flow cytometry enables the quantification of NPs’ cell uptake using their light scattering and fluorescence signals. The CeO NPs are not fluorescent, so it was necessary to conjugate a fluorescent label to impart their optical detection. FITC is a standard fluorescence label molecule, which was conjugated with the CeO and 20% Gd-CeO NPs. Figure 5 presents the absorption spectra of 0.05 M and 0.1 M CeO and 20% Gd-CeO NPs without and with FITC labelling compared to pure FITC. The FITC absorbs most efficiently at its excitation maximum at 490 nm. The absorbance peak was noticed at λ = 300 nm, corresponding to the characteristic absorption peak of Ce^4+^ of CeO_2_ NPs. Additionally, FITC labelled CeO and 20% Gd-CeO NPs exhibited bands corresponding to FITC around 490 nm as well as for Ce^4+^at 302 nm. It is the confirmation that the absorption spectrum depicts the successful loading of FITC in NPs.

**Fig. 6:**
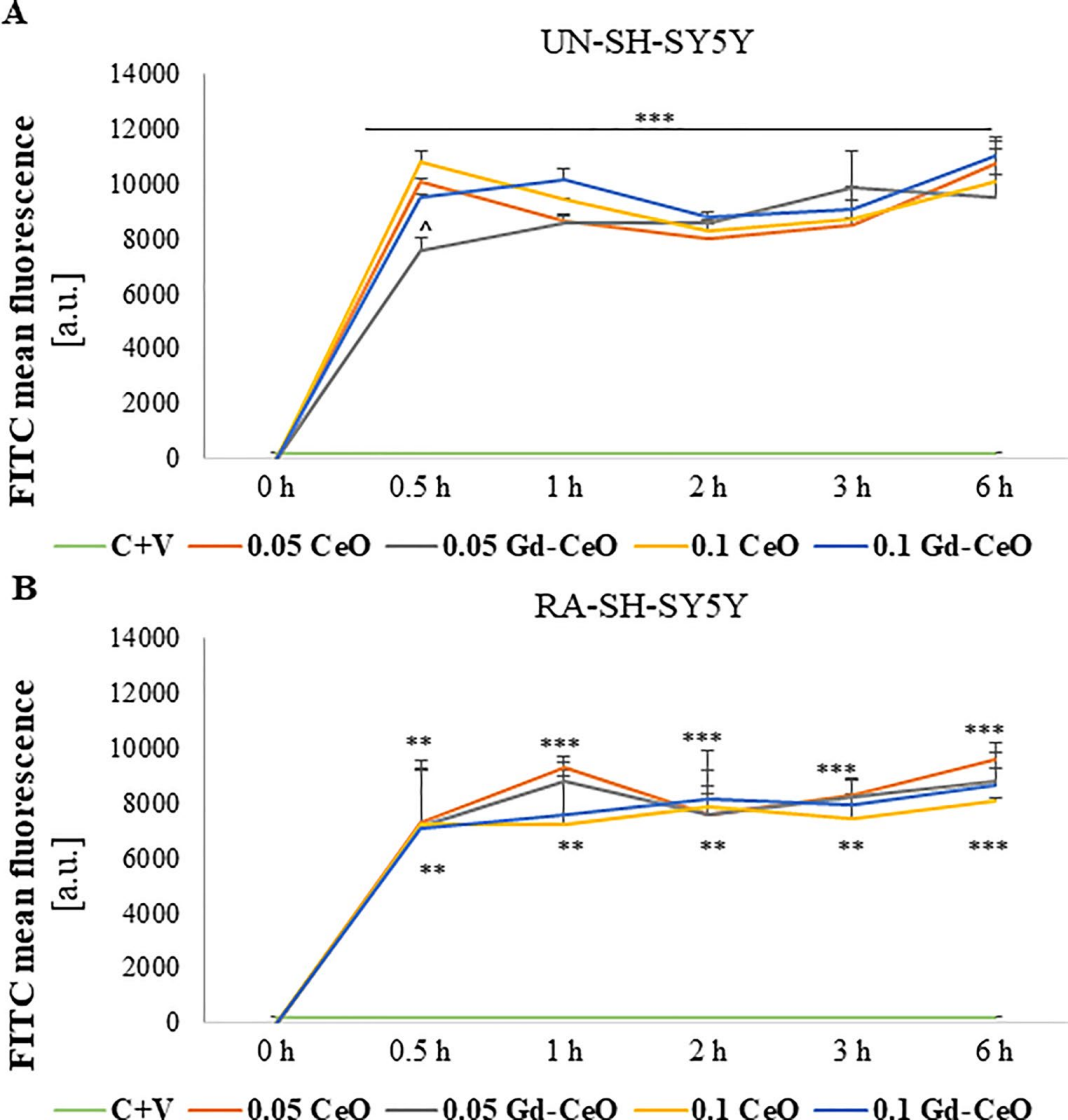
The cellular uptake of FITC-labelled NPs in UN- (**A**) and RA- (**B**) SH-SY5Y cells. The cells were incubated for 0.5 h, 1 h, 2 h, 3 h, and 6 h with vehicle (C + V; 10% v/v distilled water) or FITC-labelled 0.05 M and 0.1 M CeO and 20% Gd-CeO NPs at a dilution of 10x. The FITC mean intensity measured by flow cytometry in cells is an indicator of nanoparticle accumulation. The data are presented as a mean.±.SEM from two independent experiments; two-way ANOVA followed by Duncan post hoc; **p < 0.01 and ***p < 0.001 vs. vehicle-treated cells; ^*p* < 0.05 0.05 M Gd-CeO vs. 0.05 M CeO. C - control; CeO – polyacrylic acid-conjugated cerium oxide nanoparticles; FITC – fluorescein isothiocyanate; Gd-CeO – polyacrylic acid-conjugated cerium oxide nanoparticles; NPs – nanoparticles; V – vehicle

The flow cytometry analysis of UN- and RA-SH-SY5Y cells treated for 0.5 h, 1 h, 2 h, 3 h, and 6 h with FITC-labelled 0.05 M and 0.1 M CeO and 20% Gd-CeO NPs at a dilution of 10x demonstrated a fast cellular uptake of all NPs, as evidenced by the number of FITC-positive cells, which reached maximal signal (approx. 100%) after 0.5 h and was maintained at this level up to 6 h (data not shown). The two-way ANOVA analysis of the FITC mean intensity, an indicator of NPs cellular accumulation, demonstrated a time-dependent increase in this parameter with maximal accumulation after 0.5 h in UN- (treatment effect F_4,25_ = 245.2, p = 0.0000; time effect F_4,25_ = 4.7, p = 0.006; interaction treatment x time F_16,25_ = 1.8, p = 0.09402) (Fig. 6A) and RA-SHSY5Y cells (treatment effect F_4,25_ = 42.2, p = 0.0000; time effect F_4,25_ = 0.8, p = 0.551; interaction treatment x time F_16,25_ = 0.2, p = 0.999) (Fig. 6B). We noticed only one significant difference in NPs accumulation between CeO and Gd-doped NPs, which was found for concentration 0.05 M after 0.5 h in UN-SH-SY5Y cells where the FITC mean intensity for 0.05 M 20% Gd-CeO NPs was lower than in 0.05 M CeO NPs (Fig. 6A). In RA-SH-SY5Y cells, we did not find any differences in NPs accumulation between the tested FITC-labelled CeO NPs w/o and with Gd doping (Fig. 6B). The representative images of cellular uptake of FITC-labelled 20% Gd-CeO NPs (0.1 M) at dilution 10x after incubation for 0.5 and 6 h in UN- and RA-SH-SY5Y cells are presented in Fig. S2. Flow cytometry representative histograms for NPs cellular uptake in UN-SH-SY5Y and RA-SH-SY5Y cells are provided in Fig. S3 and Fig. S4.

### Neuroprotective effects of CeO and Gd-CeO NPs against oxidative stress-induced cell damage

**Fig. 7:**
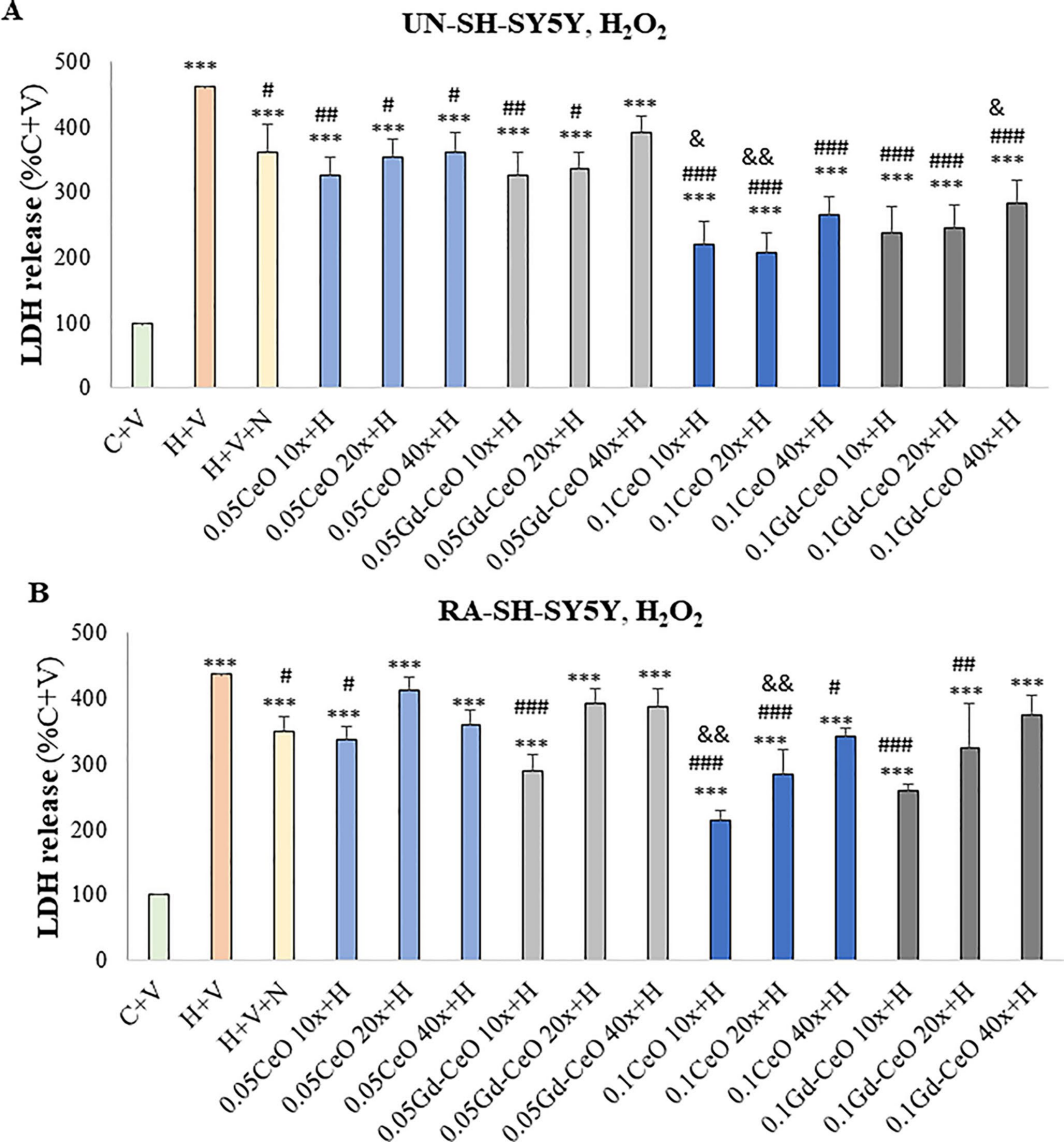
Neuroprotective effects of CeO NPs and 20% Gd-CeO NPs on cell damage induced by H_2_O_2_ in UN- (**A**) and RA- (**B**) SH-SY5Y cells. The cells were pre-treated for 30 min. with vehicle (C + V; 10% v/v distilled water), 0.05 M and 0.1 M CeO and 20% Gd-CeO NPs at dilutions 10x, 20x, and 40x followed by 24 h of treatment with H_2_O_2_ (0.375 and 0.5 mM for UN- and RA-SH-SY5Y cells, respectively). N-acetyl cysteine (N, 1 mM) given concomitantly with cell-damaging factors was used as a positive control for both oxidative stress models. Cytotoxicity was estimated by measurement of LDH level released into the cell culture medium after cell treatment. The data were normalized to vehicle-treated cells and are expressed as the mean ± SEM from 3–6 independent experiments; one-way ANOVA followed by Duncan post hoc; ^***^p < 0.001 vs. vehicle-treated cells; ^#^*p* < 0.05, ^##^*p* < 0.01, and ^###^*p* < 0.001 vs. H_2_O_2_-treated cells; ^&^*p* < 0.05 and ^&&^*p* < 0.0 0.1 M CeO or 20% Gd-CeO vs. 0.05 M CeO or 20% Gd-CeO-treated cells at particular dilutions. C - control; CeO – polyacrylic acid-conjugated cerium oxide nanoparticles; Gd-CeO – polyacrylic acid-conjugated cerium oxide nanoparticles; H – H_2_O_2_; N – NAC; NPs – nanoparticles; V – vehicle

To test the neuroprotective potential of CeO and 20% Gd-CeO NPs, the UN- and RA-SH-SY5Y cells were pretreated with different dilutions of NPs (10x, 20x, and 40x) for 30 min, followed by 24 h exposure to cell-damaging factors (H_2_O_2_ or 6-OHDA). The assessment of LDH release in the H_2_O_2_ model of UN-SH-SY5Y cells damage revealed significant differences between groups, as assessed by the one-way ANOVA (F_14,56_ = 9.8, *p* = 0.0000) (Fig. 7A). Duncan’s post hoc analysis showed that an almost 3-fold increase in cytotoxicity after incubation of cells with H_2_O_2_ (0.375 mM) was substantially reduced by positive control, NAC (1mM), all tested dilutions of 0.05 M CeO, 0.1 M CeO and 0.1 M 20% Gd-CeO NPs, and at dilution of 10x and 20x of 0.05 M 20% Gd-CeO NPs (Fig. 7A). Moreover, we found a significantly higher protection mediated by 0.1 M CeO (dilutions 10x and 20x) or 0.1 M 20% Gd-CeO NPs (dilution 40x) when compared to 0.05 M CeO or 0.05 M 20% Gd-CeO NPs at particular dilutions (Fig. 7A). It should be noted that protection mediated by dilutions 10x and 20x of 0.1 M CeO and 0.1 M 20% Gd-CeO NPs was significantly higher than the effect mediated by positive control, NAC (Fig. 7A). In RA-SH-SY5Y cells after the H_2_O_2_ (0.5 mM) and NPs exposure we found significant differences between groups as assessed by the one-way ANOVA (F_14,44_ = 14.9, *p* = 0.0000) (Fig. 7B). The H_2_O_2_ evoked over 3-fold increase in LDH release, which was significantly attenuated by NAC, 10x dilution of 0.05 M CeO, 0.05 M 20% Gd-CeO NPs, by all tested dilutions of 0.1 M CeO NPs, and by 10x and 20x dilutions of 0.1 M 20% Gd-CeO NPs (Fig. 7B). Similarly to UN-SH-SY5Y cells, we observed a concentration-dependent neuroprotective effect of NPs in RA-SH-SY5Y cells in the H_2_O_2_ model of cell damage, as evidenced by higher neuroprotection mediated by 0.1 M CeO at dilutions 10x and 20x when compared to 0.5 M CeO NPs (Fig. 7B). There was also significantly better protection mediated by 0.1 M CeO or 0.1 M 20% Gd-CeO NPs when compared to the positive control NAC (Fig. 7B).

**Fig. 8:**
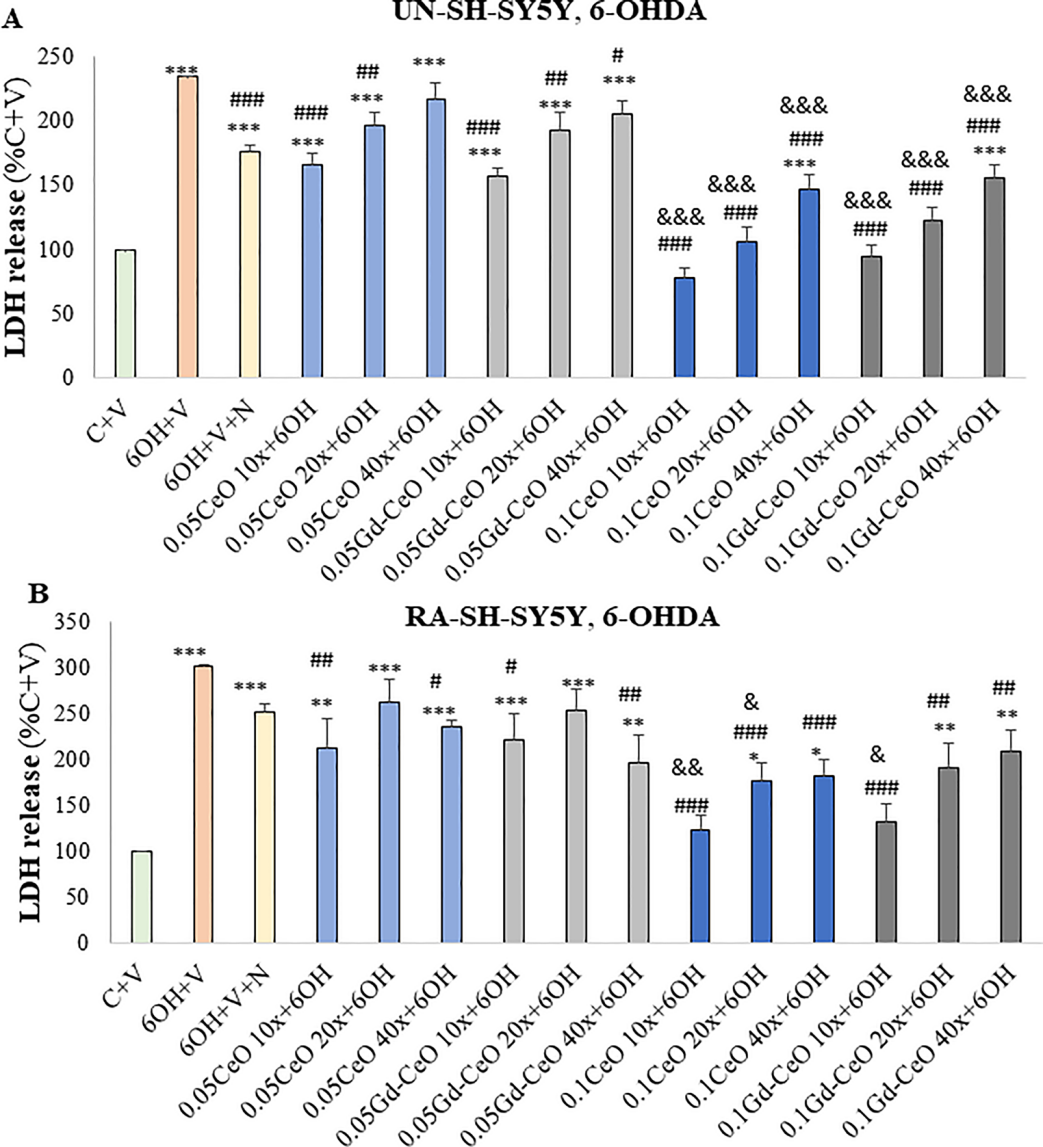
Neuroprotective effects of CeO and 20% Gd-CeO NPs on cell damage induced by 6-OHDA in UN- (**A**) and RA- (**B**) SH-SY5Y cells. The cells were pre-treated for 30 min. with vehicle (C + V; 10% v/v distilled water), 0.05 M and 0.1 M CeO and 20% Gd-CeO NPs at dilutions 10x, 20x, and 40x followed by 24 h of treatment with 6-OHDA (0.1 and 0.2 mM for UN- and RA-SH-SY5Y cells, respectively). N-acetyl cysteine (1 mM) given concomitantly with cell-damaging factors was used as a positive control for both oxidative stress models. Cytotoxicity was estimated by measurement of the LDH level released into the cell culture medium after cell treatment. The data were normalized to vehicle-treated cells and are expressed as the mean ± SEM from 4–6 independent experiments; one-way ANOVA followed by Duncan post hoc; ^*^p < 0.05, ^**^p < 0.01, and ^***^p < 0.001 vs. vehicle-treated cells; ^#^*p* < 0.05, ^##^*p* < 0.01, and ^###^*p* < 0.001 vs. 6-OHDA-treated cells; ^&^*p* < 0.05, ^&&^*p* < 0.01, and ^&&&^*p* < 0.001 0.1 M CeO or 20% Gd-CeO vs. 0.05 M CeO or 20% Gd-CeO-treated cells at particular dilutions. 6-OH – 6-OHDA; C – control; CeO – polyacrylic acid-conjugated cerium oxide nanoparticles; Gd-CeO – polyacrylic acid-conjugated cerium oxide nanoparticles; N – NAC; NPs – nanoparticles; V – vehicle

**Fig. 9:**
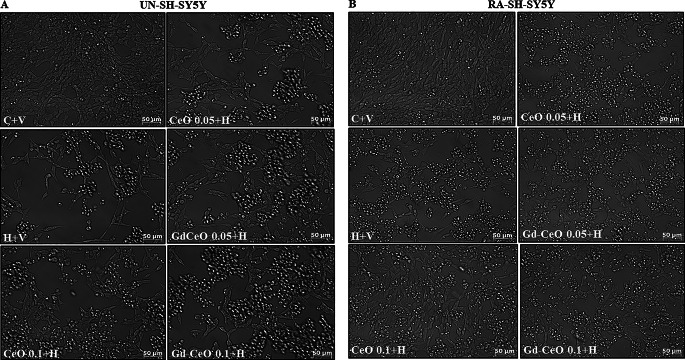
Representative DIC microphotographs of UN- (**A**) and RA- (**B**) SH-SY5Y pretreated for 30 min with vehicle (C + V; 10% v/v distilled water), 0.05 M or 0.1 M CeO and 20% Gd-CeO NPs at dilution 10x, followed by 24 h of incubation with H_2_O_2_ (0.375 and 0.5 mM for UN- and RA-SH-SY5Y, respectively). 6-OH – 6-OHDA; C – control; CeO – polyacrylic acid-conjugated cerium oxide nanoparticles; DIC (differential interference contrast); Gd-CeO – polyacrylic acid-conjugated cerium oxide nanoparticles; H – H_2_O_2_; N – NAC; NPs – nanoparticles; V – vehicle

The assessment of LDH release in the 6-OHDA model of UN-SH-SY5Y cells damage revealed significant differences between groups, as assessed by the one-way ANOVA (F_14,57_ = 32.4, *p* = 0.0000) (Fig. 8A). Post hoc analysis demonstrated that the 6-OHDA (0.1 mM)-evoked over 2-fold increase in LDH release was substantially reduced by NAC (1 mM), 0.05 M CeO NPs (by dilutions 10x and 20x) and by all tested dilutions (10-40x) of 0.05 M 20% Gd-CeO, 0.1 M CeO, and 0.1 M 20% Gd-CeO NPs (Fig. 8A). Moreover, we observed a significant concentration-dependent effect between concentrations 0.1 M and 0.05 M of CeO and 20% Gd-CeO NPs at each tested dilution (Fig. 8A). It should be noted that protection mediated by 0.05 M CeO (dilution 40x), 0.05 M 20% Gd-CeO (dilution 40x),

0.1 M CeO (all dilutions) and 0.1 M 20% Gd-CeO NPs (dilutions 10x and 20x) was significantly better than the effect of NAC (Fig. 8A). In RA-SH-SY5Y cells after the 6-OHDA (0.2 mM) and NPs exposure we found significant differences between groups as assessed by the one-way ANOVA (F_14,52_ = 7.7, *p* = 0.0000) (Fig. 8B). The 6-OHDA-evoked over 3-fold increase in LDH release was significantly attenuated by 10x and 40x dilution of 0.05 M CeO and 0.5 M 20% Gd-CeO NPs, and all dilutions (10-40x) of 0.1 M CeO and 20% Gd-CeO NPs (Fig. 8B). In addition, a significant concentration-dependent effect in neuroprotective action was found for 0.1 M CeO (for dilutions 10x and 20x) and for 0.1 M 20% Gd-CeO NPs (for dilution 10x) when compared to the relevant 0.05 M nanoparticle type and dilution (Fig. 8B). It should be noted that 0.1 M CeO (dilutions10x and 20x) and 0.1 M 20% Gd-CeO NPs (dilution 10x) were better in attenuation of the 6-OHDA-evoked LDH release in RA-SH-SY5Y cells than NAC (Fig. 8B). The protection mediated by NPs in both cell damage models (H_2_O_2_ and 6-OHDA) in UN- and RA-SH-SY5Y cells was also evidenced by light microscopy DIC imaging as shown in Fig. 9A-B and Fig. S5A-B.

We also tested the neuroprotective potential of synthesized cerium nanotheranostics after 20 months of storage in the dark at room temperature. The data from the LDH release assay after 24 h of UN-SH-SY5Y cell treatment confirmed neuroprotection by 0.05 M CeO, 0.05 M 20% Gd-CeO, 0.1 M CeO, and 0.1 M 20% Gd-CeO NPs against H_2_O_2_ (Fig. S6A; F_10,11_ = 25.4, *p* = 0.0000) or 6-OHDA (Fig. S6B, F_6,7_ = 7.6, *p* = 0.009) to a similar extent as observed with freshly prepared NPs (Figs. 7A and 8A).

### The effect of CeO and Gd-CeO NPs on necrotic markers

**Fig. 10:**
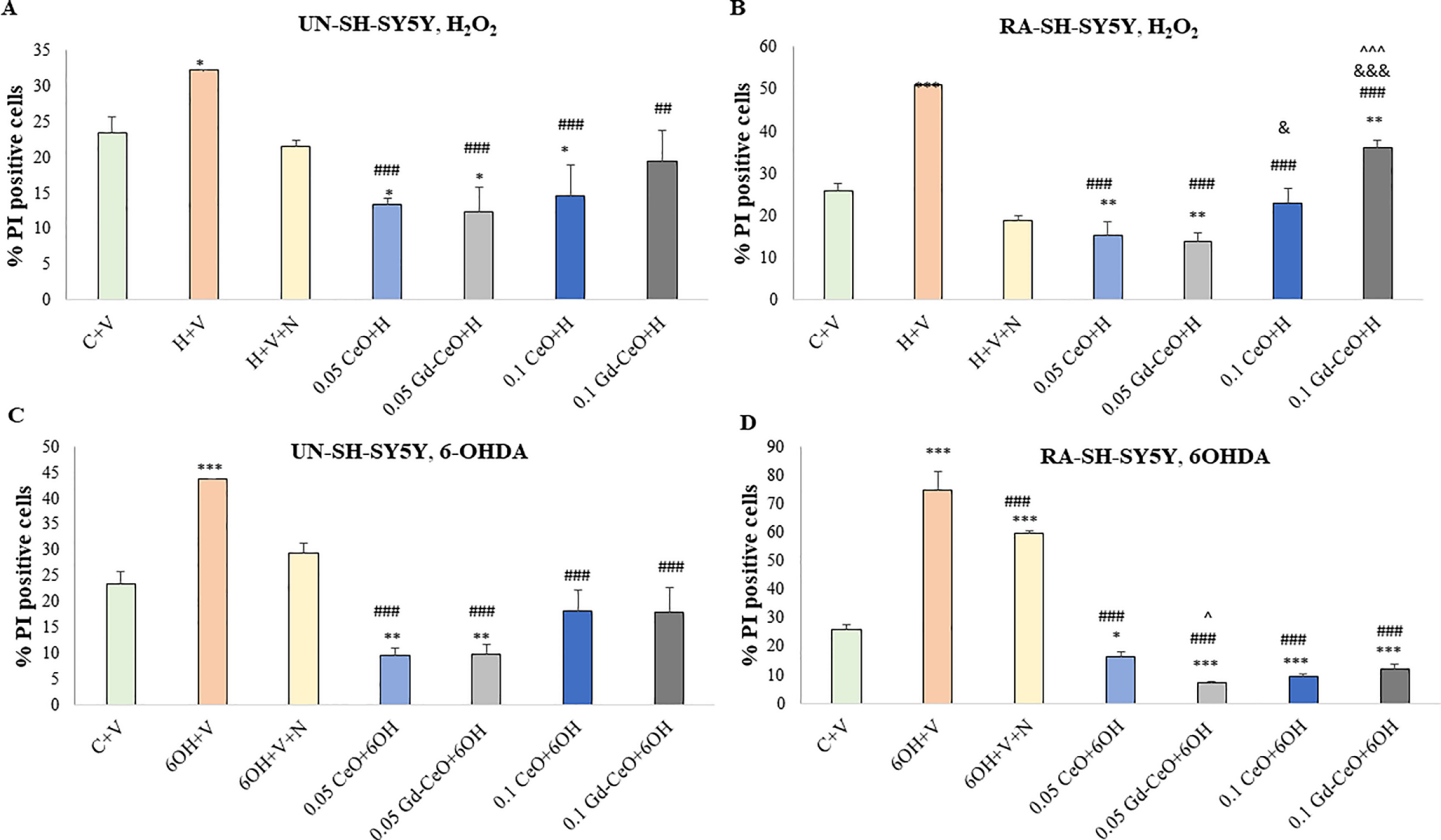
Flow cytometry analysis of PI-stained cells treated with CeO and 20% Gd-CeO NPs against H_2_O_2_ (**A**, **B**) and 6-OHDA (**C**, **D**) in UN- and RA-SH-SY5Y cells. The cells were pre-treated for 30 min. with vehicle (C + V; 10% v/v distilled water), 0.05 M and 0.1 M CeO and 20% Gd-CeO NPs at dilutions 10x, followed by 24 h of treatment with H_2_O_2_ (0.375 and 0.5 mM for UN- and RA-SH-SY5Y cells, respectively) or 6-OHDA (0.1 and 0.2 mM for UN- and RA-SH-SY5Y cells, respectively). N-acetyl cysteine (NAC, 1 mM) given concomitantly with cell-damaging factors was used as a positive control for both oxidative stress models. Cells after treatment were stained with PI and analyzed by flow cytometry. The data are expressed as the mean ± SEM of PI-positive cells from 2–3 independent experiments; one-way ANOVA followed by Duncan post hoc; ^*^p < 0.05, ^**^p < 0.01, and ^***^p < 0.001 vs. vehicle-treated cells; ^#^*p* < 0.05, ^##^*p* < 0.01, and ^###^*p* < 0.001 vs. H_2_O_2_/6-OHDA-treated cells; ^&^*p* < 0.05 and ^&&&^*p* < 0.001 0.1 M CeO or Gd-CeO vs. 0.05 M CeO or Gd-CeO-treated cells; ^^^*p* < 0.05 and ^^^^^*p* < 0.001 Gd-CeO vs. CeO for particular concentrations. 6OH – 6-OHDA, C – control, CeO – polyacrylic acid-conjugated cerium oxide nanoparticles; Gd-CeO – polyacrylic acid-conjugated cerium oxide nanoparticles; H – H_2_O_2_; N – NAC; NPs – nanoparticles; PI – propidium iodide; V – vehicle

Further, the neuroprotective potential of 0.05 and 0.1 M CeO and 20% Gd-CeO NPs in both cell phenotypes was confirmed at the level of necrotic marker, PI staining. The assessment of PI-positive cells after treatment of UN-SH- (Fig. 10).

SY5Y cells with H_2_O_2_ and NPs revealed significant differences between groups, as assessed by the one-way ANOVA (F_6,20_ = 7.1, *p* = 0.003) (Fig. 10A). Post hoc analysis revealed that CeO NPs at both concentrations (0.05 and 0.1 M) with and without Gd doping under the tested dilution 10x totally reduced the H_2_O_2_-induced increase in the number of PI-positive nuclei in UN-SH-SY5Y cells (Fig. 10A). Moreover, this protection in the case of 0.05 M 20% Gd-CeO NPs was significantly better than that of the effect mediated by the positive control, NAC (Fig. 10A). In RA-SH-SY5Y cells we found a significant effect of treatment with H_2_O_2_ and NPs as evidenced by one-way analysis of PI-positive cells (F_6,21_ = 33.9, *p* = 0.0000) (Fig. 10B). We observed a complete reduction of the H_2_O_2_-evoked increase in the number of necrotic cells by NAC, 0.05 M CeO, 0.05 M 20% Gd-CeO, and 0.1 M CeO NPs (Fig. 10B). For 0.1 M 20% Gd-CeO NPs, we demonstrated partial reduction of the H_2_O_2_-evoked increase in the number of PI-positive nuclei, which was significantly lower than that observed for its lower concentration (0.05 M 20% Gd-CeO NPs), as well as when compared to the same concentration without Gd (0.1 M CeO NPs) (Fig. 10B). The assessment of PI-positive cells after treatment of UN-SH-SY5Y cells with 6-OHDA and NPs revealed significant differences between groups, as assessed by the one-way ANOVA (F_6,21_ = 18.5, *p* = 0.0000) (Fig. 10C). We observed a complete reduction in PI-positive cells with all tested NPs, all these effects were superior to those of NAC (Fig. 10C). In RA-SH-SY5Y cells we found a significant effect of treatment with 6-OHDA and NPs as evidenced by one-way analysis of PI-positive cells (F_6,21_ = 97.0, *p* = 0.0000). Post hoc analysis showed that all NPs attenuated the toxin-induced number of necrotic cells and these effects were also better than those observed for NAC. Moreover, we found in RA-SH-SY5Y cells that 0.05 M 20% Gd-CeO NPs significantly better attenuated the 6-OHDA-induced increase in PI-positive cells when compared to 0.05 M CeO NPs (Fig. 10D). Representative flow cytometry histograms of PI staining for UN-SH-SY5Y and RA-SH-SY5Y cells are presented in Fig. S7 and Fig. S8.

### The effect of CeO and Gd-CeO NPs on caspase-3 activity

**Fig. 11:**
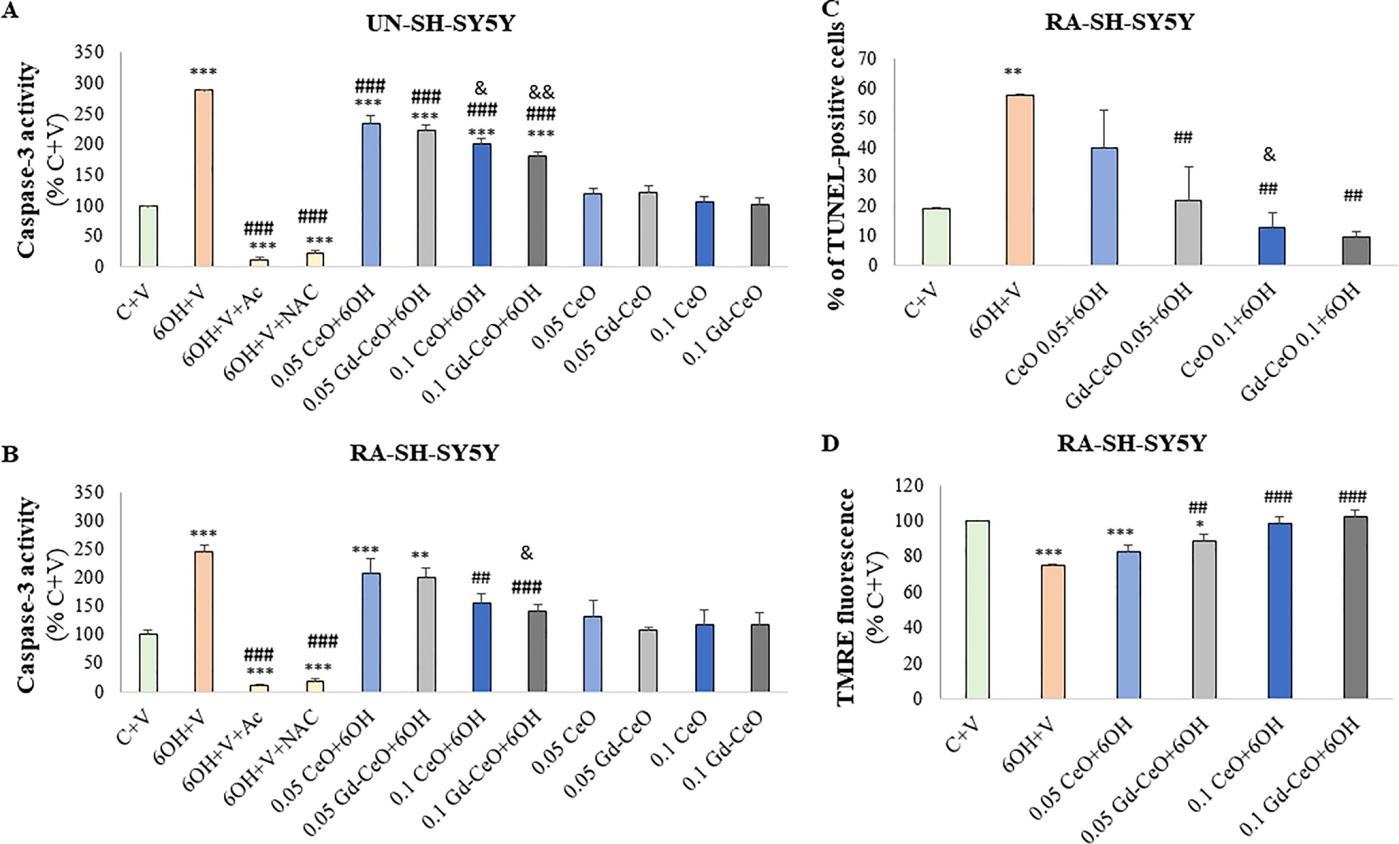
**(A-B)** The effects of CeO and 20% Gd-CeO NPs on the 6-OHDA-induced caspase-3 activity in UN- and RA-SH-SY5Y cells. The cells were pre-treated for 30 min. with vehicle (C + V; 10% v/v distilled water), 0.05 M and 0.1 M CeO and 20% Gd-CeO NPs at dilutions 10x, followed by 18 h of treatment with 6-OHDA (0.1 and 0.2 mM for UN- and RA-SH-SY5Y cells, respectively). Caspase-3 inhibitor Ac-DEVD-CHO (20 µM), given 30 min before toxin and N-acetyl cysteine (NAC, 1 mM) given concomitantly with toxin, were used as positive control for the assay. The data are normalized to vehicle-treated cells and are presented as the mean ± SEM from 3 independent experiments with two replicates. **(C-D)** The effects of CeO and 20% Gd-CeO NPs on the 6-OHDA-induced DNA fragmentation (**C**) and evoked decrease in mitochondrial membrane potential (MMP, **D**). The RA-SH-SY5Y cells were pre-treated for 30 min. with vehicle (C + V), 0.05 M and 0.1 M CeO and 20% Gd-CeO NPs at dilutions 10x, followed by 24 h (TUNEL labelling) and 6 h (MMP measurement) treatment with 6-OHDA (0.2 mM). Cells after treatment were labelled with TUNEL and analyzed by flow cytometry to assess the number of cells with fragmented DNA or stained with TMRE for measurement of changes in MMP by fluorescence microplate reader. The data for TUNEL labelling and MMP are presented as a mean percentage of TUNEL-positive cells and as a mean ± SEM, respectively from 3 independent experiments; one-way ANOVA followed by Duncan post hoc; ^*^p < 0.05, ^**^p < 0.01, and ^***^p < 0.001 vs. vehicle-treated cells; ^##^*p* < 0.01 and ^###^*p* < 0.001 vs. 6-OHDA-treated cells; ^&^*p* < 0.05 0.1 M CeO or Gd-CeO vs. 0.05 M CeO or Gd-CeO. 6OH – 6-OHDA; Ac – Ac-DEVD-CHO; C – control; CeO – polyacrylic acid-conjugated cerium oxide nanoparticles; Gd-CeO – polyacrylic acid-conjugated cerium oxide nanoparticles; N – NAC; NPs – nanoparticles; V- vehicle

Based on our previous studies, which showed partial attenuation by CeO NPs of apoptotic cell death marker caspase-3 activity in the mechanism of 6-OHDA- but not H_2_O_2_-evoked cell damage [22–23], we presently tested the effect of CeO and 20% Gd-CeO NPs on this enzyme activity in UN-and RA-SH-SY5Y cells exposed to 6-OHDA. The assessment of caspase-3 activity after treatment of UN-SH-SY5Y cells with 6-OHDA and NPs revealed significant differences between groups, as assessed by the one-way ANOVA (F_11,42_ = 88.7, *p* = 0.0000). We observed almost 3-fold the activation of caspase-3 after 18 h of treatment with 6-OHDA (0.1 mM), which was significantly attenuated by 0.5 and 0.1 M concentrations of CeO and 20% Gd-CeO NPs at the tested dilution 10x (Fig. 11A). Moreover, we observed a higher attenuation of caspase-3 activity with 0.1 M CeO and 0.1 M 20% Gd-CeO NPs compared to their lower concentrations (0.05 M) (Fig. 11A). The assessment of caspase-3 activity after treatment of RA-SH-SY5Y cells with 6-OHDA and NPs revealed significant differences between groups, as assessed by the one-way ANOVA (F_11,41_ = 14.1, *p* = 0.0000). In RA-SH-SY5Y cells, we observed almost a 2.5-fold increase in caspase-3 activity, which was attenuated by higher (0.1 M) but not lower (0.05 M) concentrations of CeO and 20% Gd-CeO NPs at the tested dilution 10x (Fig. 11B). In the case of 20% Gd-CeO NPs, we observed a significantly higher attenuation of caspase-3 activity by 0.1 M when compared to 0.05 M NPs (Fig. 11B). In both cell phenotypes, we observed a complete inhibition of 6-OHDA-induced caspase-3 activity by caspase-3 inhibitor, Ac-DEVD-CHO and NAC, and we did not find any induction of this apoptotic marker by all tested NPs when given without a cell-damaging factor (Fig. 11A-B).

### The effects of CeO and Gd-CeO NPs on DNA fragmentation

Next, the neuroprotective potential of CeO and 20% Gd-CeO NPs in RA-SH-SY5Y cells was confirmed at the level of apoptotic fragmentation of DNA measured by the TUNEL labelling method [37]. One-way ANOVA analysis of TUNEL-positive cells after H_2_O_2_ and NPs treatment showed a significant difference between the tested groups (F_5,28_ = 2.0, p = 0.104). We demonstrated about 40% of TUNEL-positive cells after treatment with H_2_O_2_ (0.5 mM) for 24 h, which was significantly attenuated by 0.1 M 20% Gd-CeO but not by 0.05 M CeO, 0.05 M 20% Gd-CeO or 0.1 M CeO NPs at the tested dilution 10x (Table 4). One-way ANOVA analysis of TUNEL-positive cells after 6-OHDA and NPs treatment showed a significant differences between tested groups (F_5,12_ = 6.0, p = 0.005) (Fig. 11C). We observed about 60% of TUNEL-positive cells after 6-OHDA treatment, which amount was significantly reduced by 10x dilution of 0.05 M 20% Gd-CeO NPs, 0.1 M CeO NPs, and 0.1 M 20% Gd-CeO NPs (Fig. 11C). Moreover, we observed a significantly higher reduction in the number of apoptotic cells by 0.1 M CeO when compared to the effect of 0.05 M CeO NPs (Fig. 11C). Representative flow cytometry histograms of TUNEL staining are presented in Fig. S10.

### The effects of CeO and Gd-CeO NPs on intracellular reactive oxygen species (ROS) level

In our previous study, we showed that the neuroprotective effects of CeO (0.03 M) or Eu-doped CeO (0.03 M) NPs against the H_2_O_2_-evoked cell damage in undifferentiated SH-SY5Y cells were not associated with the direct inhibition of intracellular ROS production [22–23]. Since we presently use higher concentrations of CeO (0.05 M and 0.1 M), it has been suggested that CeO or 20% Gd-CeO NPs could have direct ROS scavenging properties. We verified this hypothesis in RA-SH-SY5Y cells by measuring intracellular ROS production after H_2_O_2_ and 0.05 M CeO or 20% Gd-CeO NPs exposure. One-way ANOVA analysis of CM-DCF fluorescence showed a significant difference between the tested groups (F_6,13_ = 54.3, *p* = 0.0000). However, the H_2_O_2_-induced increase in the ROS level was attenuated by positive control, NAC, but not by 0.05 M CeO or 0.05 M 20% Gd-CeO NPs (Fig. S9). The 0.5 M CeO and 0.05 M 20% Gd-CeO NPs alone did not evoke a significant change in CM-DCF fluorescence compared to vehicle-treated cells, but they significantly increased the H_2_O_2_-induced increase in this parameter (Fig. S9).

### The effects of CeO and Gd-CeO NPs on mitochondrial membrane potential (MMP)

A moderate decrease in mitochondrial membrane potential is an early moment in the execution of the mitochondrial apoptotic cell death pathway. In contrast, a higher MMP collapse suggests a more necrotic phenotype of cell death [33–34]. In our study performed in RA-SH-SY5Y cells, we observed a slight reduction (about 22%) of MMP by H_2_O_2_ (0.5 mM) after 6 h of treatment, although not significant (Table 4; F_5,28_ = 2.0, *p* = 0.102). However, after combined treatment of cells with H_2_O_2_ and a higher concentration (0.1 M) of CeO or 20% Gd-CeO NPs, we found a significant increase in MMP when compared to cells treated only with this cell-damaging factor (Table 4). In the 6-OHDA model of cell damage of RA-SH-SY5Y after 6 h of treatment (F_5,42_ = 13.1, *p* = 0.0000), we observed a significant reduction in MMP (about 25%), which was significantly prevented by 10x dilution of 0.05 M 20% Gd-CeO, 0.1 M CeO, and 0.1 M 20% Gd-CeO NPs (Fig. 11D).

**Table 4:** TUNEL labelling and MMP measurement in RA-SH-SY5Y cells exposed to CeO and 20% Gd-CeO NPs and H_2_O_2_

	% of TUNEL positive cells	TMRE fluorescence [% C + V]
C + V	19.05 ± 0.19	100.00 ± 0.00
H + V	40.67 ± 0.97 ^*^	77.85 ± 0.15
0.05 M CeO + H	33.70 ± 5.09	105.42 ± 9.07
0.05 M 20% Gd-CeO + H	29.02 ± 7.27	111.84 ± 15.77
0.1 M CeO + H	28.55 ± 7.76	139.05 ± 23.92 ^#^
0.1 M 20% Gd-CeO + H	20.02 ± 5.29 ^#^	138.28 ± 25.52 ^#^

The RA-SH-SY5Y cells were pre-treated for 30 min. with vehicle (C + V, 10% v/v distilled water), 0.05 M and 0.1 M CeO, and 20% Gd-CeO NPs at dilutions 10x, followed by 24 h (TUNEL labelling) or 6 h (MMP measurement) of treatment with H_2_O_2_ (0.5 mM). Cells after treatment were labelled with TUNEL and analyzed by flow cytometry to assess the number of cells with fragmented DNA or stained with TMRE for measurement of changes in MMP by fluorescence microplate reader. The data for TUNEL labelling and MMP are presented as a mean percentage of TUNEL-positive cells and as a mean ± SEM, respectively from 3 independent experiments; one-way ANOVA followed by Duncan post hoc; ^*^p < 0.05 vs. vehicle-treated cells; ^#^*p* < 0.05 vs. H_2_O_2_-treated cells. C – control; CeO – polyacrylic acid-conjugated cerium oxide nanoparticles; Gd-CeO – polyacrylic acid-conjugated cerium oxide nanoparticles; H - H_2_O_2_; MMP – mitochondrial membrane potential; TMRE – tetramethylrhodamine ethyl ester perchlorate; TUNEL – terminal deoxynucleotidyl transferase (TdT)-mediated dUTP nick-end labeling; V- vehicle

### The effect of CeO and Gd-CeO NPs on cathepsin D (Cth D) activity

Since our previous studies demonstrated an involvement of cathepsin D activation in detrimental effects of H_2_O_2_ in UN- and RA-SH-SY5Y cells [32, 37], we also measured this enzyme activity in the present study. In UN-SH-SY5Y cells, we observed a significant effect of overall treatment as showed by one-way ANOVA analysis (F_11,24_ = 4.4, *p* = 0.001), with only a small, not significant increase in CthD activity (about 30%), which was significantly reduced by CthD inhibitor - PsA, but not by the tested NPs. The tested NPs when given alone did not evoke significant increase in CthD activity, but there could be observed some tendency for activation by 0.1 M CeO NPs (*p* = 0.110) and after combined treatment of UN-SH-SY5Y cells with H_2_O_2_ at a 10x dilution of 0.05 M CeO, 0.05 M 20% Gd-CeO, 0.1 M CeO, or 0.1 M 20% Gd-CeO NPs, we observed a significant activation of CthD but only when compared to vehicle-treated cells (Table 5). In RA-SH-SY5Y cells, we did not find any increase in CthD activity after 18 h of treatment with H_2_O_2_, or after combined treatment with the tested NPs (F_11,24_ = 4.2, *p* = 0.002). Nevertheless, we noticed a significant reduction in this enzyme activity after PsA treatment and some tendency for activation for 0.05 M Gd-CeO (*p* = 0.135) and 0.1 M CeO NPs (*p* = 0.101) when given alone (Table 5).

**Table 5:** The effect of CeO and 20% Gd-CeO NPs on the H_2_O_2_-induced Cathepsin D activity in UN- and RA-SH-SY5Y cells

	UN-SH-SY5Y	RA-SH-SY5Y
C + V	100.00 ± 0.00	100.00 ± 0.00
H + V	130.63 ± 5.82	97.58 ± 8.08
0.05 M CeO + H	163.14 ± 7.75 ^*^	107.18 ± 5.53
0.05 M 20% Gd-CeO + H	158.50 ± 18.52 ^*^	103.59 ± 5.66
0.1 M CeO + H	166.69 ± 23.46 ^*^	111.09 ± 3.71
0.1 M 20% Gd-CeO + H	160.43 ± 26.46 ^*^	108.19 ± 5.89
H + PsA	44.92 ± 9.82 ^* ##^	53.21 ± 26.89 ^* #^
0.05 M CeO	131.86 ± 11.41	120.12 ± 8.86
0.05 M 20% Gd-CeO	126.31 ± 9.38	124.03 ± 6.50
0.1 M CeO	143.09 ± 18.36	126.64 ± 4.81
0.1 M 20% Gd-CeO	127.43 ± 14.94	115.96 ± 3.30

The UN- and RA-SH-SY5Y cells were pre-treated for 30 min. with vehicle (C + V, 10% v/v distilled water), 0.05 M and 0.1 M CeO and 20% Gd-CeO NPs at dilutions 10x, alone or followed by 18 h treatment with H_2_O_2_ (0.375 and 0.5 mM for UN- and RA-SH-SY5Y cells, respectively). Pepstatin A (0.3 µM) was used as a positive control for the assay. Cathepsin activity was measured in cell lysates using fluorogenic substrate AMC-Gly-Lys-Pro-Ile-Leu-Phe-Phe-Arg-Leu-Lys(Dnp)-D-Arg-NH2. The data were normalized to vehicle-treated cells and are expressed as the mean ± SEM from 3 independent experiments; one-way ANOVA followed by Duncan post hoc; *p < 0.05 vs. vehicle-treated cells; ^#^*p* < 0.05 and ^##^*p* < 0.01 vs. H_2_O_2_-treated cells. C – control, CeO – polyacrylic acid-conjugated cerium oxide nanoparticles; Gd-CeO – polyacrylic acid-conjugated cerium oxide nanoparticles; H - H_2_O_2_; NPs – nanoparticles; PsA – pepstatin A; V- vehicle

### The effect of CeO and Gd-CeO NPs on calpain activity

Since in our previous studies we showed the involvement of activation of calpains, calcium-dependent intracellular proteases, in the mechanisms of H_2_O_2_-evoked neuronal cell damage in SH-SY5Y cells [37], we measured the level of spectrin α II total (148–204 kDa) and its cleavage products induced by calpains (101–131 kDa) or caspases (83–112 kDa) with quantitative ProteinSimple Jess method. Although, one-way ANOVA analysis of total spectrin α II did not reveal significant changes between experimental groups (F_8,9_ = 2.0, *p* = 0.155) (Fig. S11A), we observed a significant changes between tested experimental groups in calpain (F_8,9_ = 3.4, *p* = 0.043) (Fig. S11B) and caspase (F_8,9_ = 9.6, *p* = 0.001) (Fig. S11C) mediated spectrin α II cleavage products. However, we did not find any attenuation by 0.05 M CeO or 0.05 M 20% Gd-CeO NPs of the H_2_O_2_- or 6-OHDA-evoked increase in calpain (Fig. S11B) or caspases (Fig. S11C) spectrin α II cleavage products. The NPs alone (0.05 M) did not affect the total or calpain or caspase-related spectrin α II cleavage products (Fig. S11A-C). Portrayal of a traditional blot-like image and total protein measurement for tested samples are presented in Fig. S11 D-E.

### The effects of CeO and Gd-CeO NPs against H_2_O_2_- or glutamate-induced cell damage in primary neuronal cell cultures

**Fig. 12:**
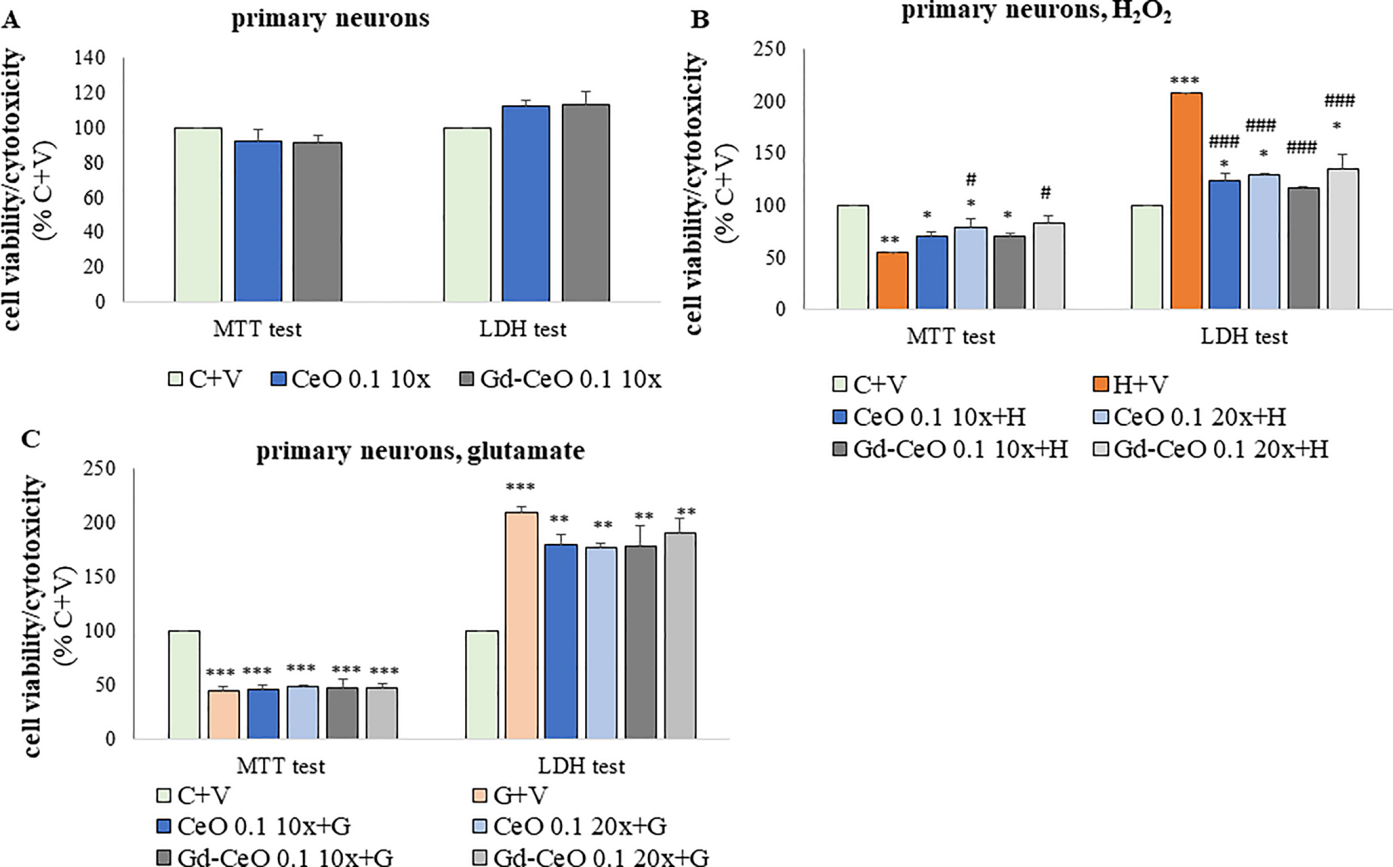
The effects of CeO and 20% Gd-CeO NPs alone (**A**) and on cell damage induced by H_2_O_2_ (**B**) or glutamate (**C**) in primary cortical neurons. The cells were treated for 24 h or pre-treated for 30 min. with vehicle (C + V; 10% v/v distilled water), 0.1 M CeO and 0.1 M 20% Gd-CeO NPs at dilutions 10x, followed by 24 h of treatment with H_2_O_2_ (0.125 mM) or glutamate (1 mM). Cell viability and cytotoxicity were estimated by MTT reduction and LDH release assays, respectively. The data were normalized to vehicle-treated cells and are expressed as the mean ± SEM from 2 independent experiments; one-way ANOVA followed by Duncan post hoc; ^*^p < 0.05, ^**^p < 0.01 and ^***^p < 0.001 vs. vehicle-treated cells; ^#^*p* < 0.05 and ^###^*p* < 0.001 vs. H_2_O_2_-treated cells. C – control, CeO – polyacrylic acid-conjugated cerium oxide nanoparticles; G – glutamate; Gd-CeO – polyacrylic acid-conjugated cerium oxide nanoparticles; H – H_2_O_2_; NPs – nanoparticles; V – vehicle

Twenty four hours of treatment with 0.1 M CeO and 0.1 M 20% Gd-CeO NPs at dilution 10x did not evoke any detrimental effect in primary neurons as confirmed by MTT reduction (F_2,15_ = 1.1, *p* = 0.355) and LDH release (F_2,15_ = 3.0352, *p* = 0.078) assays (Fig. 12A). The assessment of LDH release and MTT reduction in the H_2_O_2_ model of neuronal cell damage revealed significant differences between groups, as assessed by the one-way ANOVA (F_5,6_ = 38.5, *p* = 0.0002 and F_5,6_ = 7.6, *p* = 0.014, respectively) (Fig. 12B). Post hoc analysis demonstrated that the H_2_O_2_-evoked LDH increase was significantly attenuated by both tested dilutions (10x and 20x) of 0.1 M CeO and 0.1 M 20% Gd-CeO NPs (Fig. 12B, right panel). However, the H_2_O_2_-induced reduction in cell viability measured by MTT test was modestly, but significantly, attenuated by 20x dilution of 0.1 M CeO and 0.1 M 20% Gd-CeO NPs; for dilution 10x of both types of NPs, we observed only some tendency for neuroprotection (*p* < 0.12) (Fig. 12B, left panel). The assessment of LDH release and MTT reduction in the Glu model of neuronal cell damage revealed significant differences between groups, as assessed by the one-way ANOVA (F_5,6_ = 12.8, *p* = 0.004 and F_5,6_ = 23.6, *p* = 0.0000, respectively) (Fig. 12B). However, after post hoc analysis, we did not observe any protection mediated by both tested dilutions of 0.1 M CeO and 0.1 M 20% Gd-CeO NPs against neuronal cell damage induced by Glu, as verified by LDH release and MTT assays (Fig. 12C).

## Discussion

In this study, the Gd-CeO NPs were successfully synthesized by doping 20% concentrations of Gd^3+^ in PAA-conjugated CeO NPs through a chemical precipitation technique. Without the conjugation of additional molecular moieties, these NPs typically display nonselective biodistribution and poor colloidal stability. Therefore, their surface properties must be modified through the conjugation of specific functional groups, polymers, or biomolecules to address these limitations. PAA is a non-toxic, biocompatible, and biodegradable polymer that has gained significant interest in recent years [38]. NPs produced with PAA as a stabilizer can be used to deliver drugs due to their stability and biocompatibility. PAA and its nanoconjugates could also be regarded as pH-responsive platforms that make them ideal for drug delivery and antimicrobial applications. These properties make PAA a good candidate for conventional and novel drug carrier systems [38]. In our previous work [22], we demonstrated the neuroprotective effects of PAA-conjugated CeO NPs against neuronal cell damage induced by oxidative stress inducers (H_2_O_2_ and 6-OHDA), which were associated with the inhibition of necrotic or apoptotic processes. We also designed Eu^3+^-doped PAA conjugated CeO NPs (Eu-CeO) to develop a theranostic agent that imparts luminescence and possesses neuroprotective activity [23]. In this study, we developed the new Gd-CeO NPs in the size range of 40–50 nm for 0.05 M Gd-CeO NPs and 20–30 nm for 0.1 M Gd-CeO NPs with a zeta potential in water of about − 40 mV in both cases. These NPs can be used as a theranostic agent with MRI capabilities and neuroprotective properties. We showed that the obtained suspensions of Gd-CeO NPs in water possessed good stability, exhibiting minimal aggregation for up to 20 months, making them suitable for biomedical applications. We also demonstrated that, through a simple modification of the synthesis process, it is possible to obtain doped CeO NPs with a wide range of biomedical applications, which is both highly innovative and practical. There is little information in the literature regarding straightforward syntheses of lanthanide-doped CeO with PAA, which further highlights the significance of our work. It should be noted that the positive MRI contrast obtained from 20% Gd-CeO NPs in our experiment was detectable up to a 64–128 dilution factor, starting from the nominal 0.1 M-0.05 M concentrations. This should make it possible to use it in vivo as a positive contrast/theranostic agent, especially with sufficient accumulation. The r_1_ value of 1,3 − 1,7 mM^-1^s^-1^ is lower than reported by Popov et al. [29], where they found r_1_ values between 2 and 3 mM^-1^s^-1^, for a magnetic field of 9-10T, which corresponds to our experimental conditions. This is most probably caused by the bio-functionalisation of Gd-CeO with PAA, which decreases magnetic interactions of Gd with water molecules, thus decreasing effective relaxation effects. However, based on the magnetic field dependence of r_1_ reported by Popov et al. [29], it may be expected that for a clinically relevant magnetic field of 1.5-3T, r_1_ will be higher. In general, Gd is considered a positive contrast agent in MRI due to the predominant effect of decreasing T_1_ relaxation time [39]. However, often some effects of decreasing T_2_ relaxation time are observed, which is also the case in our results. The r_2_ values of 8.6–11.4 mM^-1^s^-1^ are, however, very low as per other negative contrast/theranostic agents, especially based on SPIONS [40–41], where values of 600–800 mM^-1^s^-1^ are not unusual. Typically, the relaxation properties of the complexes with the paramagnetic center, are governed by direct interactions of protons from the first layer of water molecules, with the Gd^3+^ ions, as described by SBM inner-sphere model [42–44]. Proton exchange with bulk water increases T₁ shortening, enhancing r₁ relaxivity. These interactions are dependent on the number of inner-sphere water molecules directly bound to Gd^3+^, residence time of a water molecule in the inner sphere, and rotational correlation time i.e., how fast the nanoparticle or molecule tumbles. The relaxation mechanism in the outer-sphere is based on dipole–dipole interactions as water molecules diffusing near, but not directly bound to Gd³⁺. As described in the Freed model of outer-sphere relaxation, it depends on the diffusion coefficient of water, the approach distance between water and Gd^3+^ center, and the characteristic diffusion time during which water molecules pass the paramagnetic center. The interplay between all these parameters leads to the resulting value of the overall relaxivity. In our case, the important component of the molecular structure of the Gd-doped nanoparticle is the presence of the PAA coating, which thickness and stiffness define water diffusing conditions in the outer-sphere, as well as parameters influencing inner-sphere interactions. We compared our MRI data for Gd-CeO with a clinical MRI agent (e.g., Gd-DTPA) for benchmarking contrast performance. Typical values for r1 of Gd-DTPA (Magnevist) measured at 9.4T as reported in the literature are ~ 3.0 mM-1s-1 [45]. Gd-DTPA, due to its linear complex structure, is presently of very limited use due to the safety concerns (i.e., detection of Gd deposition in the brain after multiple injections). Instead, we have checked relaxation properties of a sample macrocyclic compound, namely Gd-DO3A (Gadovist), which at our experimental conditions, has r1 equal to 4.4–4.7 mM-1s-1. The obtained r1 values in our study are indeed lower than the corresponding values for clinical, gadolinium-based contrast agents, which are optimized for the image contrast effects while maintaining an appropriate level of safety. However, we were investigating contrasting properties of a potential theranostic agent (Gd-CeO), where contrasting effect is an addition to the therapeutic (neuroprotective) one. The scope of the MRI – related investigations was limited to the assessment of contrasting properties, which we found reasonable in the case of positive contrast based on T_1_ relaxation. The possible reason for the relatively low value of r1, can be attributed to the effect of the PAA coating. Other issues, e.g., stability and Gd leaching, especially under physiological conditions require further studies.

When evaluating the biosafety of synthesized 20% Gd-CeO NPs in comparison to the effect of CeO without doping it should be noted that all tested NPs were safe for undifferentiated and neuronally differentiated SH-SY5Y cells at both studied concentrations (0.05 M and 0.1 M) after 24 h, as confirmed by cytotoxicity (Table 3) and caspase-3 activity (Fig. 11A, B) assays. Moreover, we showed a very efficient cellular uptake of the FITC-labeled NPs in SH-SY5Y cells without any inhibitory influence of Gd (Fig. 6). Interestingly, in the frame of the investigated time point for cellular uptake (up to 6 h), we did not observe any decrease in FITC signal, meaning that NPs are stable and are accumulated in cells. These data support the biocompatibility of PAA-conjugated CeO (0.03 M) NPs for dopaminergic cells observed in our previous studies [22–23], although in this research, we extended these data to the higher concentrations of CeO NPs (0.05 M and 0.1 M). One could speculate that such accumulation will prevent in vivo uses of the NPs due to potential long-term cytotoxicity. Thus, more detailed studies should be done concerning the internalization of NPs and their degradation. Nevertheless, our Ce-based theranostic NPs could be regarded as safe when compared to previous data showing the cytotoxic effects of gadolinium oxide (Gd_2_O_3_) NPs, which are applied in industrial products (additives, optical glass, and catalysis) [46]. The cell damage induced by 50 and 100 µg/ml Gd_2_O_3_ NPs was recorded when given for 24 and 48 h to SH-SY5Y cells, and this effect was connected with ROS formation, mitochondrial collapse, and induction of apoptosis [46]. It is not excluded that the potential cytotoxic effect of Gd in our NPs could be masked by the presence of PAA. This assumption could be supported by other reports, where, for example, dual-functionalized Gd@C82 NPs up to 400 µg/ml were not cytotoxic when given for 24 h to primary mouse cortical neurons or astrocytes, or to SH-SY5Y cells [47].

When evaluating the neuroprotective effectiveness of synthesized Gd-doped CeO NPs in comparison to the effect of CeO NPs, it should be noted that, in general, we observed a similar neuroprotective potency of both types of NPs against cell damage induced by oxidative stress inducers, H_2_O_2_ or 6-OHDA. These results not only confirm our previous observations on neuroprotective features of PAA-conjugated CeO NPs at concentration 0.03 M alone or with Eu^3+^ doping in SH-SY5Y cells [22–23] but also extend them to higher concentrations studied (0.05 M and 0.1 M) and demonstrate a concentration-dependent neuroprotection in both studied cell damage models (H_2_O_2_ and 6-OHDA). Whereas in our previous study, we showed significantly better protection mediated by Eu-CeO against the cell damage induced by 6-OHDA in RA-SH-SY5Y cells [23], in the present study, we did not notice such clear relationships with the presence of Gd. One exception is data from the PI method, where we observed in RA-SH-SY5Y cells a significantly better attenuation of H_2_O_2_-induced necrosis by 0.1 M CeO NPs when compared to the effects of 0.1 M 20% Gd-CeO NPs (Fig. 10B); however, in the 6-OHDA model, we found better efficacy of 0.05 M 20% Gd-CeO than 0.05 M CeO (Fig. 10D). The above suggests that specific methods could be used to detect a cell-damage model-dependent, potentially detrimental or beneficial presence of Gd in NPs for neuroprotection. When it comes to the possible mechanisms of neuroprotection mediated by 20% Gd-CeO NPs, we demonstrated the involvement of inhibition of necrosis in both tested models of cell damage (Fig. 10) and inhibition of caspase-3 in 6-OHDA model (Fig. 11A, B), which is in line with our previous observations made for CeO NPs or Eu-CeO NPs [22–23]. Our ROS measurements (Fig. S9) also confirm our previous observations that CeO NPs did not attenuate ROS production in its protection against the H_2_O_2_-evoked cell damage [22–23] and extend them also to the Gd-doped CeO system. In this study, we showed for the first time for CeO NPs and their derivatives additional protective parameters like improvement of MMP and attenuation of DNA fragmentation (Fig. 11C, D, and Table 4). This is a significant observation, taking into account a crucial role of mitochondrial dysfunction in neurodegenerative diseases [48–49], and suggests that the protection by CeO NPs appears rather at early stages of cell death induction and consequently is also detected at its lower stages, like DNA fragmentation or cell membrane rupture. It will be crucial in the future to study the effects of NP on mitochondrial ROS, ATP production, mitochondrial morphology or respiratory chain activity. We also excluded the participation of inhibition of calpains, a calcium-dependent proteases in protection mediated by CeO and Gd-CeO NPs against H_2_O_2_ and 6-OHDA, measured by expression of spectrin α II cleavage product by quantitative Western blot method (ProteinSimpleJess, Fig. S11). It should be noted that in our preliminary studies we also did not observe any inhibitory effect of 0.05 M and 0.1 M CeO or 20% Gd-CeO NPs on calpains activity induced by H_2_O_2_ (data not shown) as measured by spectrin α II 145 kDa level by classical Western blot method [37]. In addition, in the present study, we also excluded the involvement of inhibition of CthD, a lysosomal enzyme engaged in the pathogenesis of various ND [50–51], in the neuroprotective effect of CeO or 20% Gd-CeO NPs against the H_2_O_2_-evoked cell death (Table 5). The observed increase in CthD activity after combined NPs treatment with H_2_O_2_ in UN-SH-SY5Y cells, as well as some tendency for induction of this enzyme by NPs alone, suggests the possible involvement of lysosomes in NPs elimination, an effect demonstrated for other types of NPs [52–53]. However, it is not excluded that this increase in CthD activity could also be a signal of lysosomal stress or early cell damage, which should be verified in future experiments. It should be taken into account that cathepsins, in dependence on expression level and disease context, could have both protective and pathological roles [50]. Among the limitations of our present work is the use of one neuronal cell line in most studies. It should be noted that we employed human neuroblastoma SH-SY5Y cells, which are a widely used cellular model for PD, and they have a dopaminergic phenotype, and express tyrosine hydroxylase, dopamine transporter DAT, or dopamine receptors in both, undifferentiated as well as differentiated phenotypes. Differentiation of cells, for example, with retinoic acid inhibits proliferation, increases neurites length, and induces intracellular pro-survival pathways (PI3-K/Akt, MAPK/ERK1/2) [54–56]. Thus, UN- and RA-SH-SY5Y as neuronal models have pros and cons, thus, in our study, we are using both cell phenotypes. In case of undifferentiated cells, the major limitation with respect to neuronal phenotype is their tumor origin and high proliferation rate, thus observed changes could be a result of the impact of the tested compounds on proliferation rather than cell death mechanism. On the other hand, differentiation of SH-SY5Y could mask the potential protective response of the tested compound. In our study, in both cell phenotypes we observed the protection by CeO or Gd-CeO against oxidative stress inducers (H_2_O_2_ and 6-OHDA), which suggests an involvement of other mechanisms than activation of pro-survival pathways like MAPK/ERK1/3 or PI3-K/Akt. Thus, the neuroprotective potential of developed Gd-CeO NPs should also be verified in the future in other neuronal in vitro models of PD, including testing their potency on alpha-synuclein aggregation, an important pathological factor of PD [57]. To this end, it has been shown that some type of Gd-based NPs (e.g., Gd@C82 NPs) could inhibit Aβ aggregation and prevent Aβ-cytotoxicity in neuronal cells [47]. However, before testing the effectiveness of synthesized NPs in animal studies of PD or other ND [58–59], it will be crucial to investigate their BBB penetrance and dopaminergic or other neuronal phenotype specificity in various cellular systems. It is highly probable that for specific brain region targeting with Gd-CeO NPs will be crucial to put “molecular address” on their surface, which will deliver them to their final destination to diagnose and treat particular ND. To this end, we confirmed the neuroprotective effects of synthesized NPs against H_2_O_2_-evoked cell damage in primary cortical neurons, however they were not effective against cell damage induced by the excitotoxic factor, glutamate (Fig. 12). This suggests the preferential neuroprotective utility of CeO or Gd-CeO NPs in oxidative stress mediated CNS pathologies, which should be confirmed in the future also in primary dopaminergic neurons and hiPSCs-derived dopaminergic neurons, as well as in animal PD models.

## Conclusions

In this study, we successfully doped PAA-conjugated CeO NPs with Gd (Gd-CeO) to create a theranostic agent with MRI capabilities and neuroprotective properties. The synthesized Gd-CeO NPs were stable and demonstrated good T_1_ (i.e., positive) contrast features, and were rapidly taken up by human neuronal-like cells (UN- and RA-SH-SY5Y cells) without showing any significant cytotoxic effects. The Gd-CeO NPs maintained neuroprotective potency against H_2_O_2_- and 6-OHDA-induced cell damage to a similar extent as did CeO NPs without Gd doping. Moreover, we demonstrated a protective effect of Gd-CeO and CeO NPs on mitochondrial membrane potential, DNA fragmentation, and the number of necrotic cells in both models of cell injury, whereas at the level of caspase-3 activity, we showed an inhibitory effect of the studied NPs only in the 6-OHDA model. Finally, the protection mediated by Gd-CeO and CeO NPs against H_2_O_2_ was confirmed in mouse primary cortical neurons. Since the developed Gd-CeO NPs maintained biosafety and neuroprotective properties similar to NPs without Gd doping, they could be further investigated as a potential theranostic probe for various ND management, including testing in more physiologically relevant cellular systems or in animal PD models.

## Electronic Supplementary Material

Below is the link to the electronic supplementary material.

Supplementary Material 1

## Data Availability

The data presented in this study are available on request from the corresponding author.

## Abbreviations

6-OHDA: 6-hydroxydopamine
BBB: The blood-brain barrier
Ce: Cerium
CeO NPs: Cerium oxide nanoparticles
CeO: Polyacrylic acid-conjugated cerium oxide nanoparticles
Cth D: Cathepsin D
DIC: Differential interference contrast
DLS: Dynamic Light Scattering
FITC: Fluorescein isothiocyanate
Gd: Gadolinium
Gd-CeO: Polyacrylic acid-conjugated cerium oxide nanoparticles with gadolinium
Glu: Glutamate
H_2_O_2_: Hydrogen peroxide
LDH: Lactate dehydrogenase
MADLS: Multi-Angle Dynamic Light Scattering
MMP: Mitochondrial membrane potential
MRI: Magnetic resonance imaging
ND: Neurodegenerative diseases
NPs: Nanoparticles
PAA: Polyacrylic acid
PD: Parkinson’s disease
PDI: Polydispersity index
PI: Propidium iodide
RA-SH-SY5Y: Retinoic acid-differentiated SH-SY5Y cells
ROS: Reactive oxygen species
SEM: Standard error of the mean
TEM: Transmission electron microscope
TMRE: Tetramethylrhodamine ethyl ester perchlorate
TUNEL: Terminal deoxynucleotidyl transferase (TdT)-mediated dUTP nick-end labeling
UN-SH-SY5Y: Undifferentiated SH-SY5Y cells
