# Supplementary material for: The impact of gadolinium doping on physicochemical properties and neuroprotective activity of polyacrylic acid conjugated cerium oxide nanoparticles – in vitro study of potential theranostics for neurodegenerative diseases
Source: Pharmacol Rep. 2026 May 9;78(4):1119–44. doi: 10.1007/s43440-026-00863-5 (PMC13437749; doi:10.1007/s43440-026-00863-5)
Supplement: Supplementary file 1 [file 43440_2026_863_MOESM1_ESM.pdf]

Supplementary Information

The impact of gadolinium doping on physiochemical properties and neuroprotective activity of polyacrylic acid conjugated cerium oxide nanoparticles – in vitro study of potential theranostics for neurodegenerative diseases

Rugmani Meenambal[1,†], Tomasz Kruk[2], Piotr Warszyński[2], Natalia Łopuszyńska[3], Władysław P. Węglarz[3], Katarzyna Stan-Głowińska[4], Joanna Wojewoda-Budka[4], Krzysztof Jasiński[3], Aleksandra Mąsior[1] and Danuta Jantas[1*]

[1]Maj Institute of Pharmacology, Polish Academy of Sciences, Smętna 12, Kraków 31-343, Poland

[2]Jerzy Haber Institute of Catalysis and Surface Chemistry, Polish Academy of Sciences, Niezapominajek 8, Kraków 30-239, Poland

[3]Henryk Niewodniczański Institute of Nuclear Physics, Polish Academy of Sciences, Radzikowskiego 152, Kraków 31-342, Poland

[4]Institute of Metallurgy and Materials Science, Polish Academy of Sciences, Reymonta 25, Kraków 30-059, Poland

*Corresponding Author: Danuta Jantas, jantas@if-pan.krakow.pl (ORCID: 0000-0002-6789-4489)

†Present Address: Quro Health and Lifestyle Private Limited, Thiruvananthapuram, India

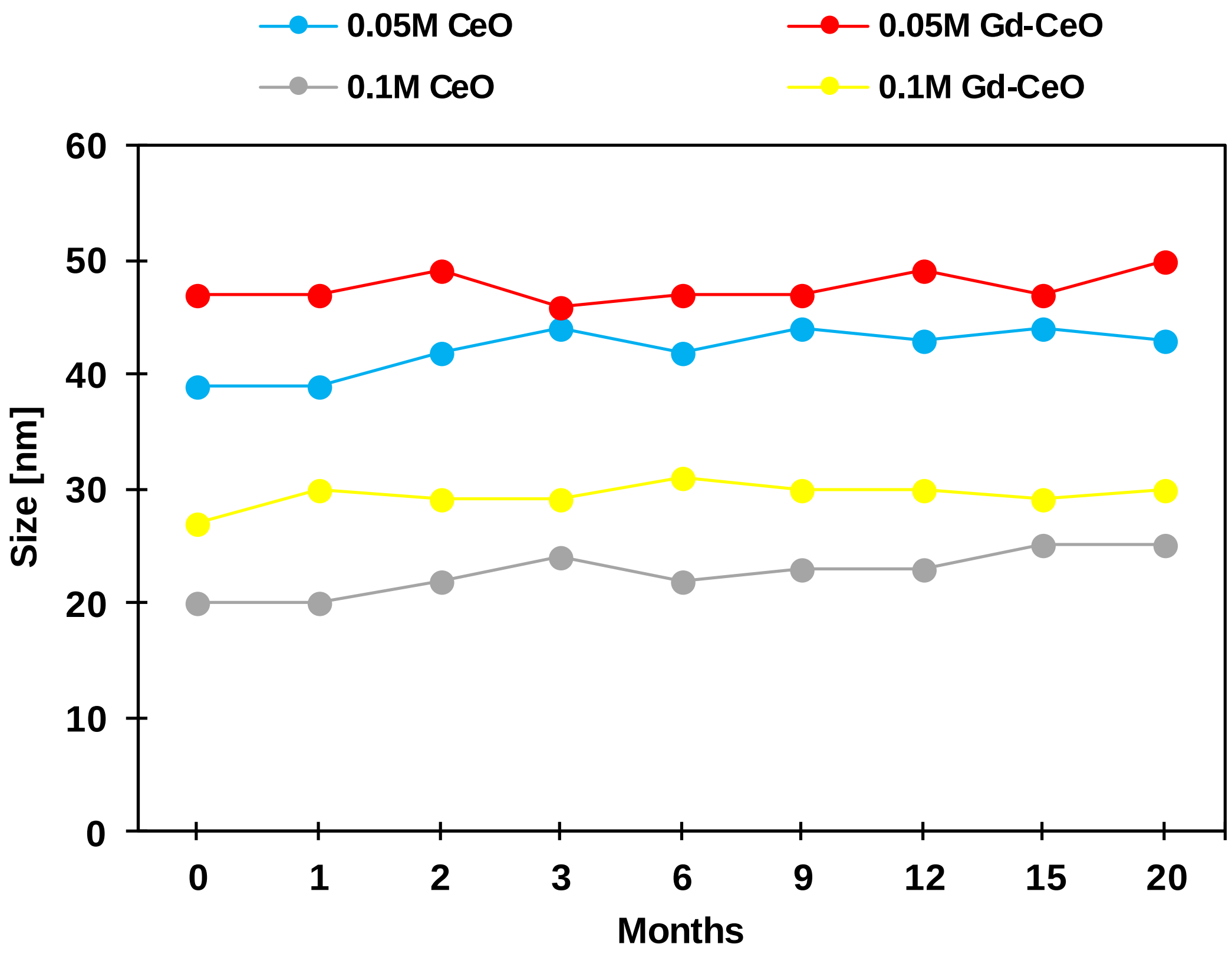

Fig. S1. The size distributions for 0.05M and 0.1M CeO and 20% Gd-CeO NPs over the time 1-20 months. CeO – polyacrylic acid-conjugated cerium oxide nanoparticles; Gd-CeO – polyacrylic acid-conjugated cerium oxide nanoparticles; NPs – nanoparticles.

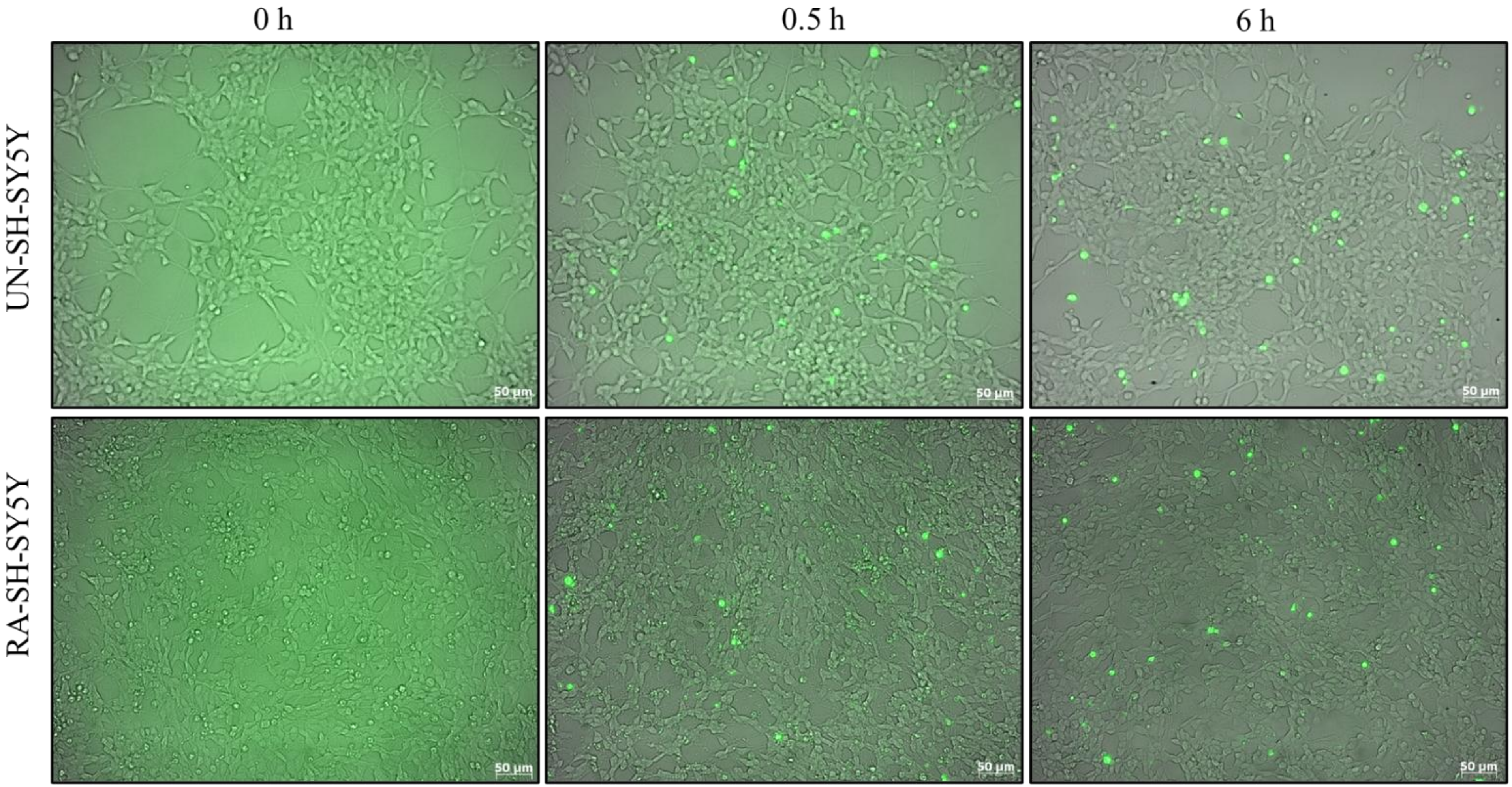

Fig. S2. Representative merged images (green and DIC channels) of cellular uptake of FITC-labelled 0.1M 20% Gd-CeO (polyacrylic acid-conjugated cerium oxide nanoparticles with gadolinium) NPs at dilution 10x after incubation for 0.5 and 6 h in UN- (upper panel) and RA- (bottom panel) SH-SY5Y cells. Images indicated as 0 h represent vehicle (10% v/v distilled water)-treated cells.

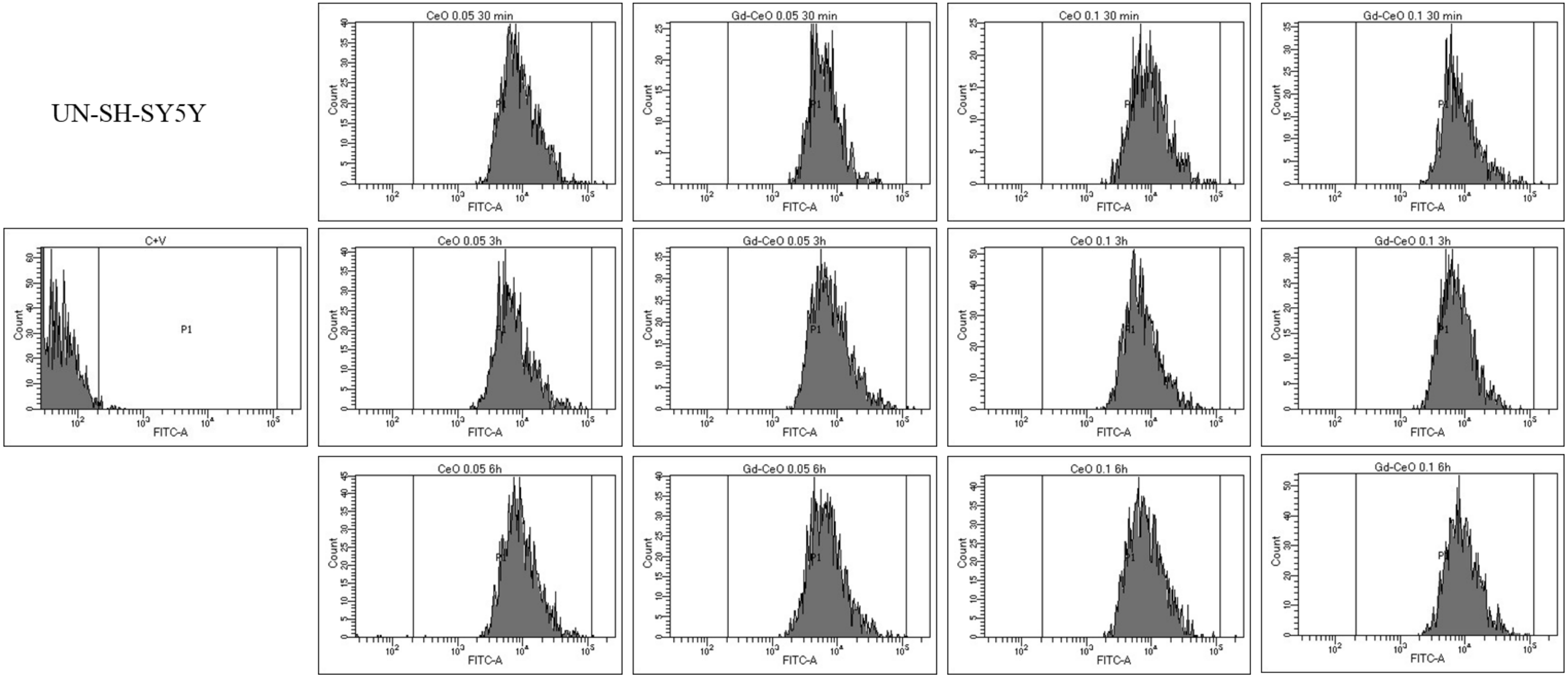

Fig. S3. Representative flow cytometry histograms of cellular uptake of FITC-labelled 0.05M and 0.1M CeO and 20% Gd-CeO NPs at dilution 10x after incubation for 30 min, 3 h and 6 h in UN-SH-SY5Y cells. Group C+V indicates cells treated with vehicle (10% v/v distilled water) and represents start point (0 h). CeO – polyacrylic acid-conjugated cerium oxide nanoparticles; FITC – fluorescein isothiocyanate; Gd-CeO – polyacrylic acid-conjugated cerium oxide nanoparticles; NPs – nanoparticles.

RA-SH-SY5Y

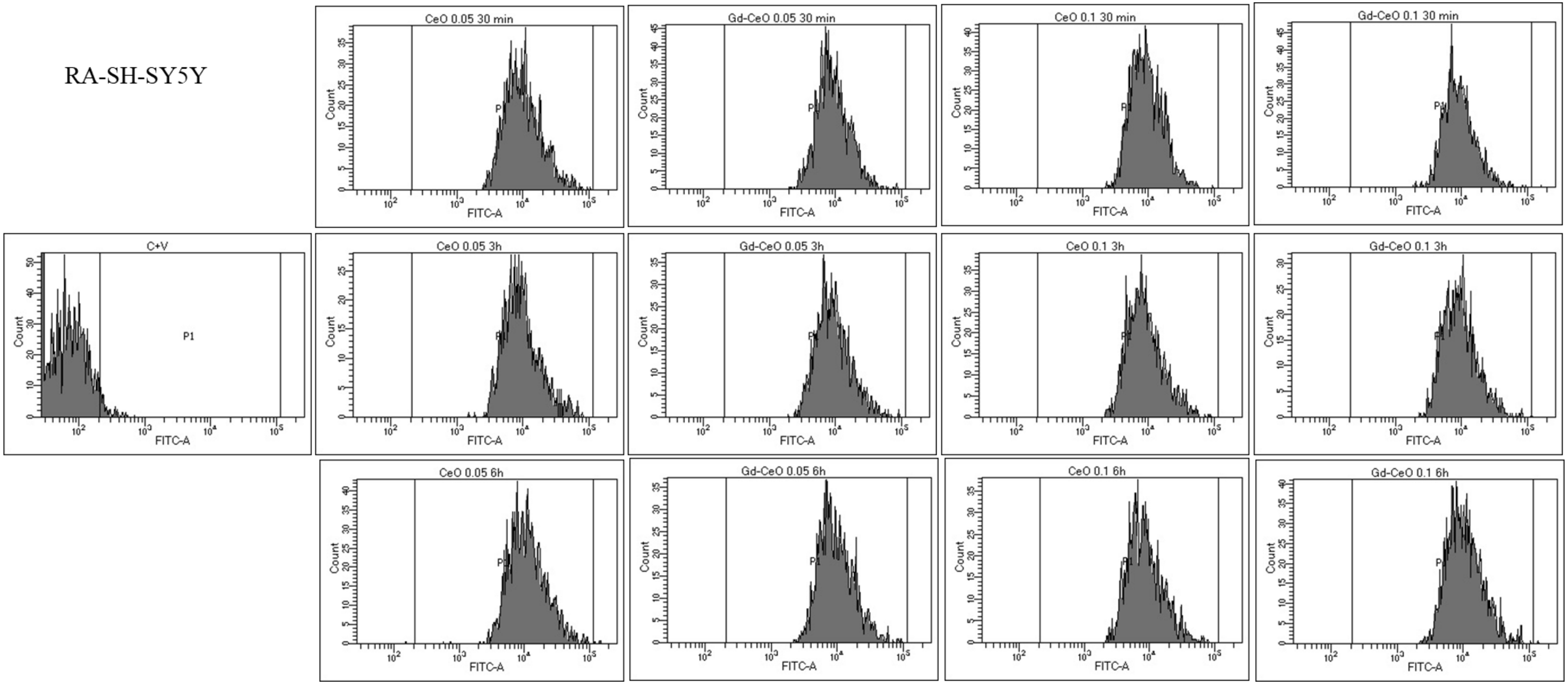

Fig. S4. Representative flow cytometry histograms of cellular uptake of FITC-labelled 0.05M and 0.1M CeO and 20% Gd-CeO NPs at dilution 10x after incubation for 30 min, 3 h and 6 h in RA-SH-SY5Y cells. Group C+V indicates cells treated with vehicle (10% v/v distilled water) and represents start point (0 h). CeO – polyacrylic acid-conjugated cerium oxide nanoparticles; FITC – fluorescein isothiocyanate; Gd-CeO – polyacrylic acid-conjugated cerium oxide nanoparticles; NPs – nanoparticles.

A

UN-SH-SY5Y

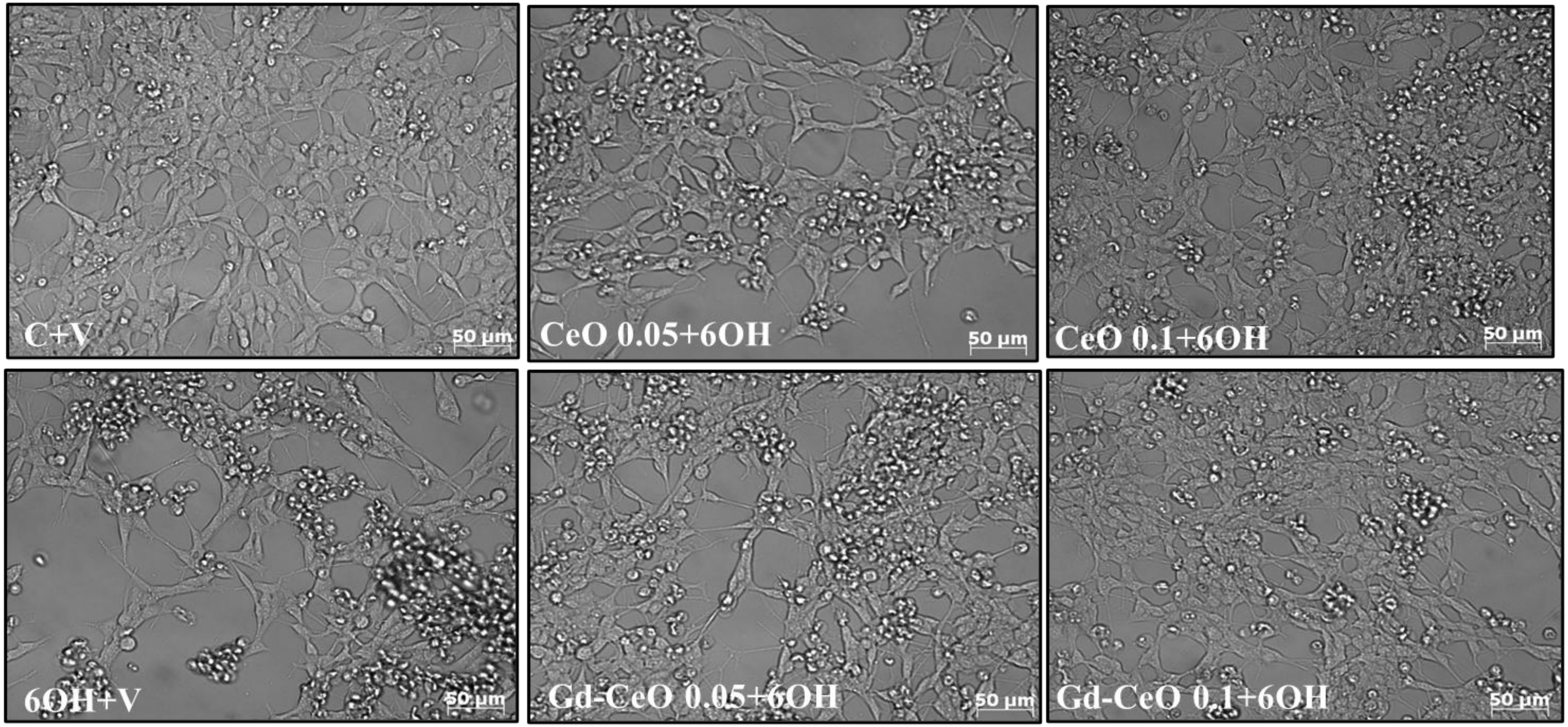

C+V
50 µm
CeO 0.05+6OH
50 µm
CeO 0.1+6OH
50 µm
6OH+V
50 µm
Gd-CeO 0.05+6OH
50 µm
Gd-CeO 0.1+6OH
50 µm

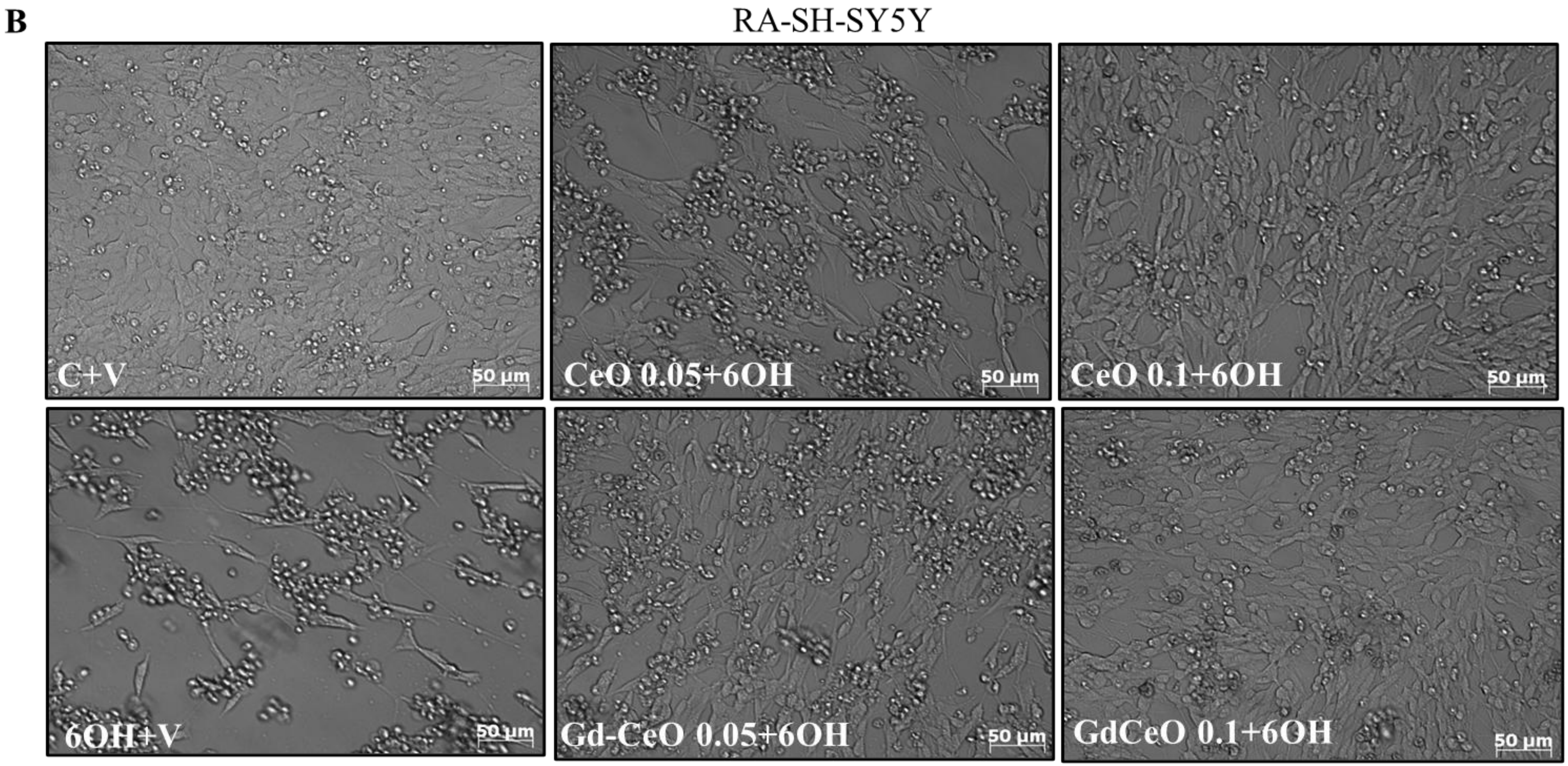

Fig. S5. Representative DIC microphotographs of UN- (**A**) and RA- (**B**) SH-SY5Y pretreated for 30 min with vehicle (10% v/v distilled water), 0.05M or 0.1M CeO and 20% Gd-CeO NPs at dilution 10x followed by 24 h of incubation with 6-OHDA (0.1 and 0.2 mM for UN- and RA-SH-SY5Y, respectively). 6OH – 6-OHDA, 6-hydroxydopamine; C – control; CeO – polyacrylic acid-conjugated cerium oxide nanoparticles; DIC – differential interference contrast); Gd-CeO – polyacrylic acid-conjugated cerium oxide nanoparticles; NPs – nanoparticles; V – vehicle.

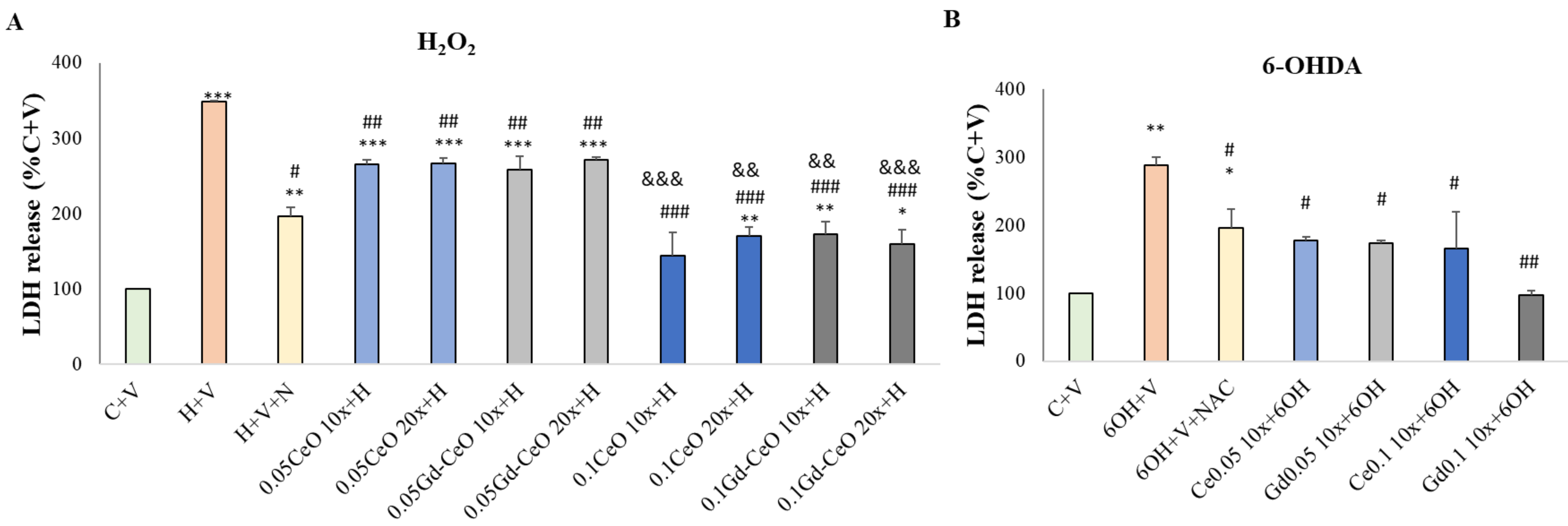

Fig. S6. Neuroprotective effects of long-term stored CeO (polyacrylic acid-conjugated cerium oxide nanoparticles) and 20% Gd-CeO (polyacrylic acid-conjugated cerium oxide nanoparticles with gadolinium) NPs on cell damage induced by H_2O_2 (A) and 6-OHDA (B) in UN-SH-SY5Y cells. The cells were pre-treated for 30 min. with vehicle (10% v/v distilled water), and stored for 20 months at room temperature and darkness 0.05M and 0.1M CeO and 20% Gd-CeO NPs at dilutions 10x and 20x followed by 24 h of treatment with H_2O_2 (0.375 mM) or 6-OHDA (0.1 mM). N-acetyl cysteine (N, 1 mM) given concomitantly with cell damaging factors was used a positive control for both oxidative stress models. Cytotoxicity was estimated by measurement of LDH level released into cell culture medium after cell treatment. The data are expressed as the mean ± SEM from 2 independent experiments; one-way ANOVA followed by Duncan posthoc; $^{*}p<0.05$, $^{**}p<0.01$ and $^{***}p<0.001$ vs. vehicle-treated cells; $^{\#}p<0.05$, $^{\#\#}p<0.01$ and $^{\#\#\#}p<0.001$ vs. H_2O_2/6-OHDA-treated cells; $^{\&\&}p<0.01$ and $^{\&\&\&}p<0.001$ 0.1M CeO or 20% Gd-CeO vs. 0.05M CeO or 20% Gd-CeO-treated cells at particular dilutions. 6-OH – 6-OHDA, 6-hydroxydopamine; C – control; CeO – polyacrylic acid-conjugated cerium oxide nanoparticles; Gd-CeO – polyacrylic acid-conjugated cerium oxide nanoparticles; H – H_2O_2; N – NAC; NPs – nanoparticles; V – vehicle.

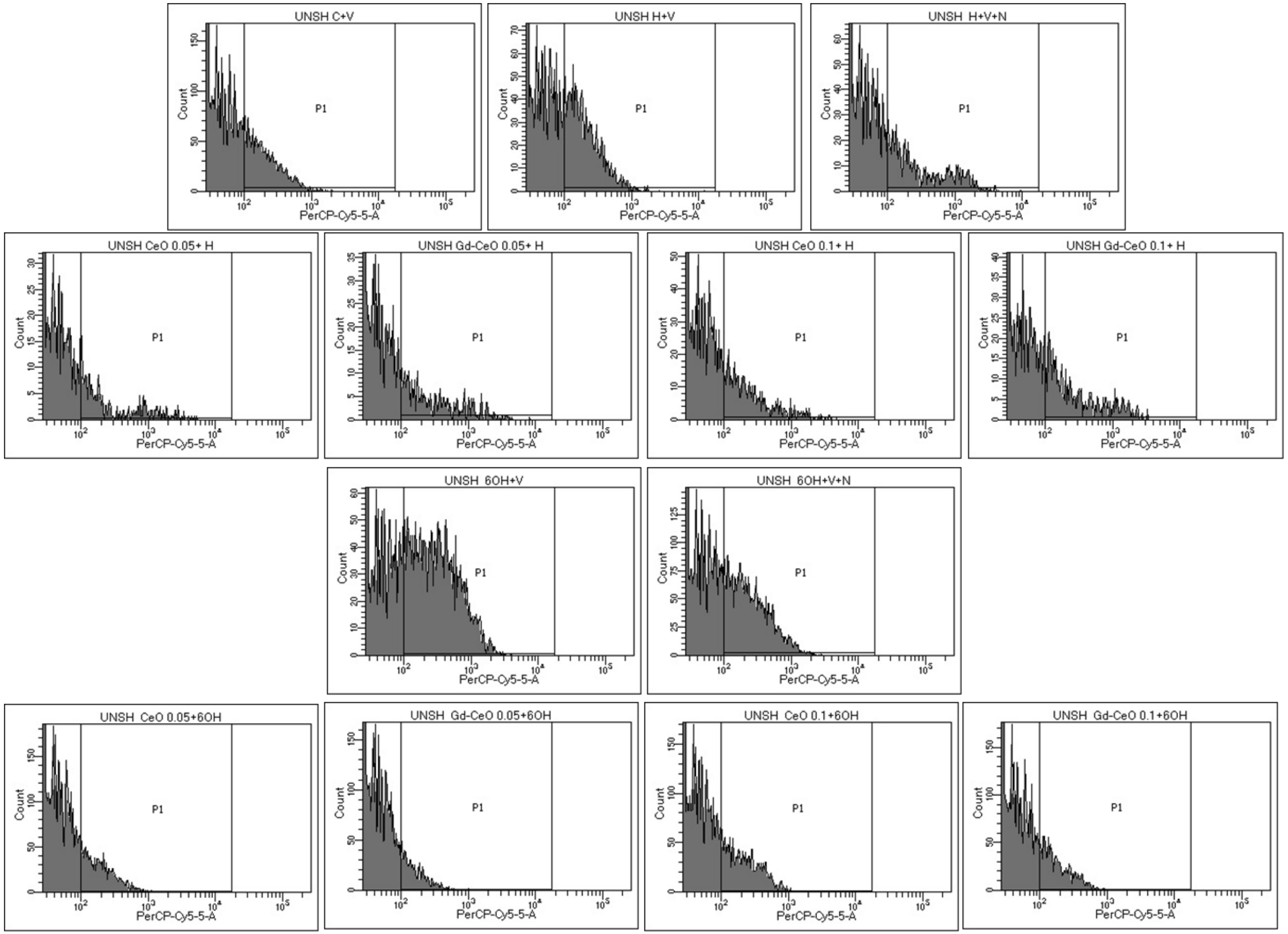

Fig. S7. Representative flow cytometry histograms of PI staining (PerCP-Cy5 channel) for UN-SH-SY5Y cells pretreated for 30 min with vehicle (10% v/v distilled water), 0.05M and 0.1M CeO and 20% Gd-CeO NPs at dilution 10x followed by 24 h treatment with H_2O_2 (0.375 mM) or 6-OHDA (0.1 mM). N-acetyl-cysteine (NAC, 1 mM) was used as a positive control. 6-OH – 6-OHDA; CeO – polyacrylic acid-conjugated cerium oxide nanoparticles; Gd-CeO – polyacrylic acid-conjugated cerium oxide nanoparticles; H – H_2O_2; N – NAC; NPs – nanoparticles; V – vehicle.

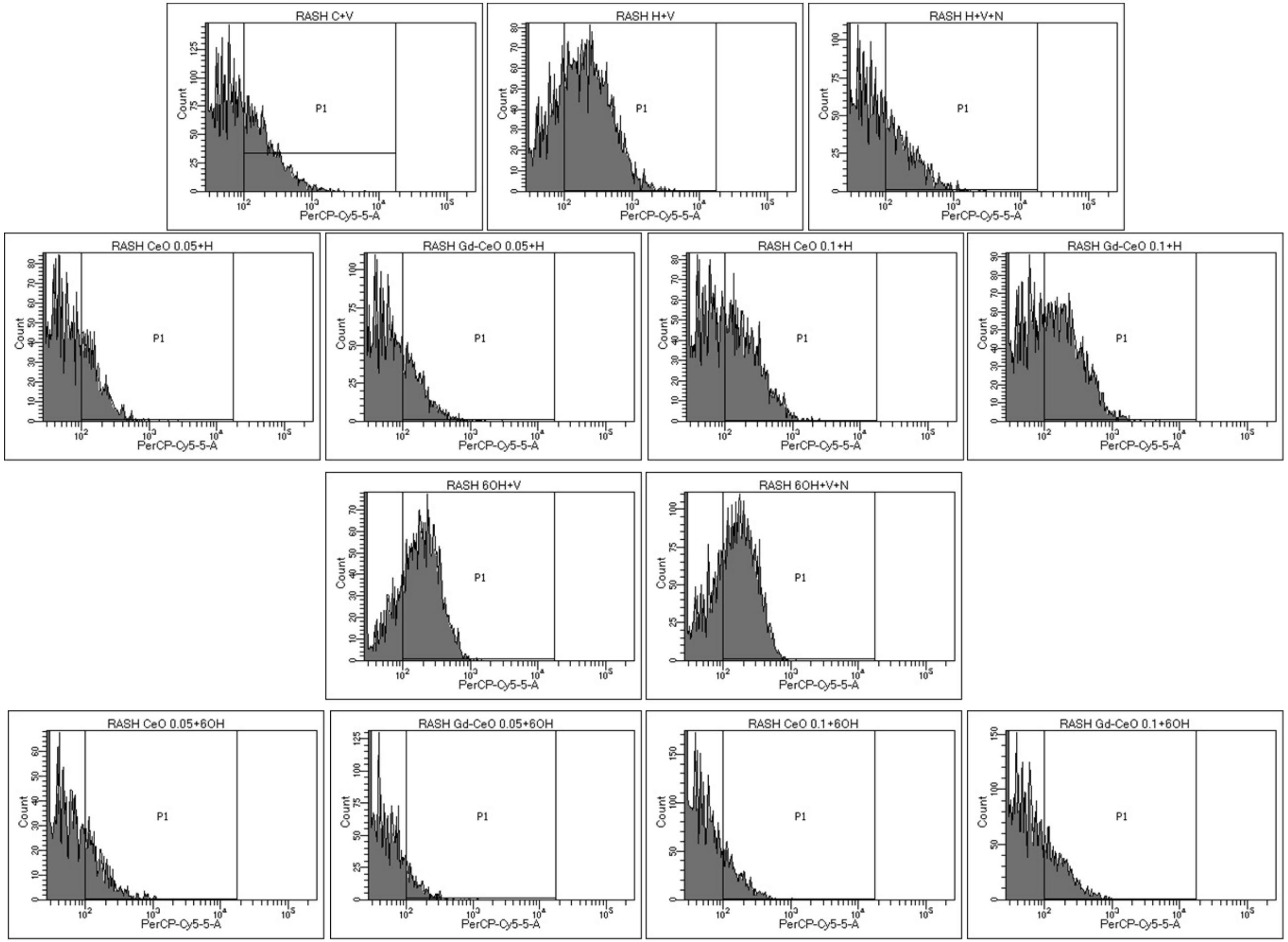

Fig. S8. Representative flow cytometry histograms of PI staining (PerCP-Cy5 channel) for RA-SH-SY5Y cells pretreated for 30 min with vehicle (10% v/v distilled water), 0.05M and 0.1M CeO and 20% Gd-CeO NPs at dilution 10x followed by 24 h treatment with H_2O_2 (0.5 mM) or 6-OHDA (0.2 mM). N-acetyl-cysteine (NAC, 1 mM) was used as a positive control. 6-OH – 6-OHDA; CeO – polyacrylic acid-conjugated cerium oxide nanoparticles; Gd-CeO – polyacrylic acid-conjugated cerium oxide nanoparticles; H – H_2O_2; N – NAC; NPs – nanoparticles; V – vehicle.

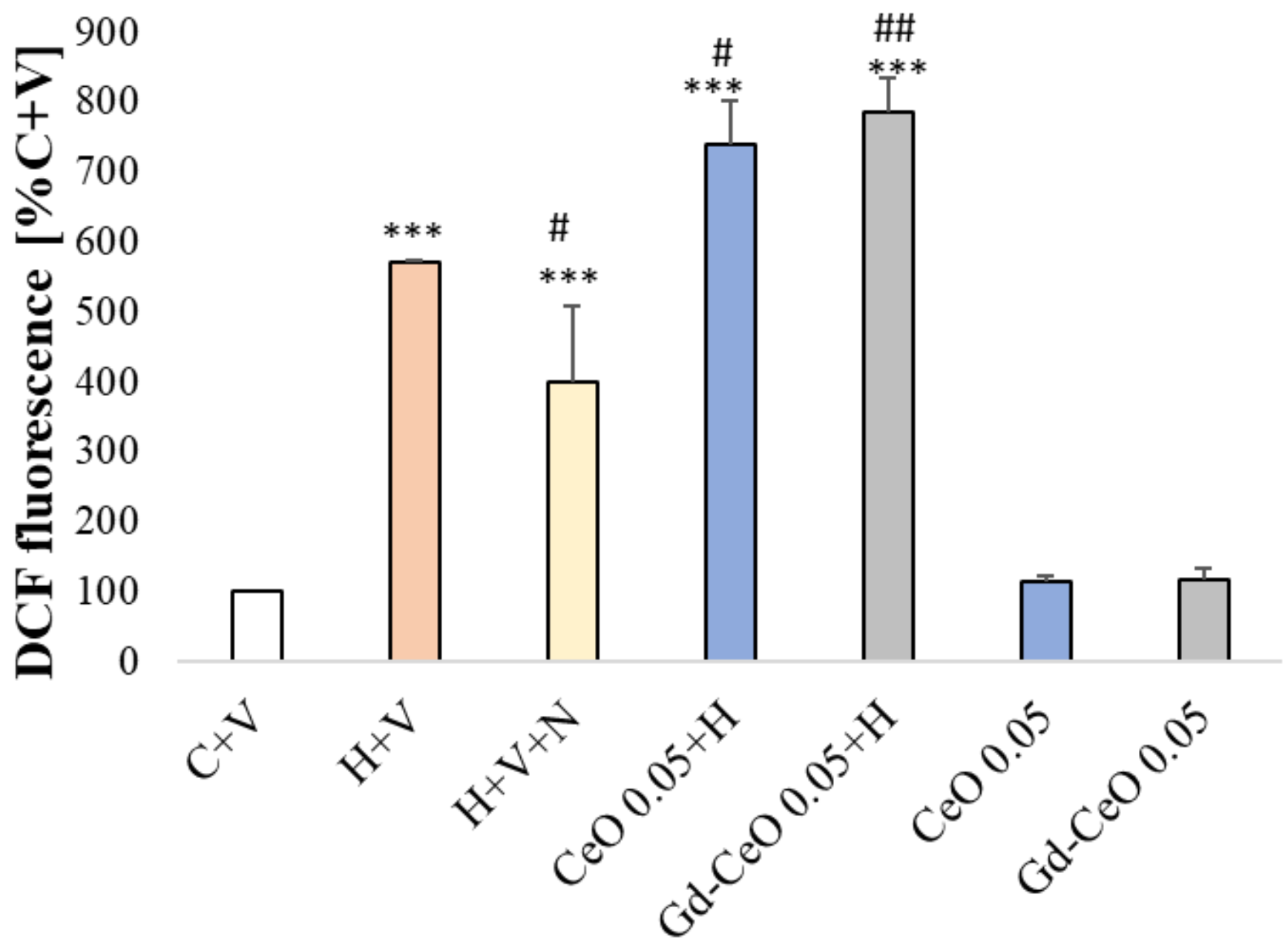

Fig. S9. The effect of CeO and 20% Gd-CeO NPs on the H_2O_2-induced increase in intracellular reactive oxygen species (ROS) level in RA-SH-SY5Y cells. The cells loaded with CM-H_2DFFDA probe (5 μM) were pre-treated for 30 min. with vehicle (10% v/v distilled water), 0.05M CeO and 20% Gd-CeO NPs at dilutions 10x followed by 30 min exposure to H_2O_2 (1 mM). N-acetyl cysteine (NAC, 1 mM) given concomitantly with H_2O_2 was used a positive control. The CM-DCF fluorescence intensity was measured in probes with multi-well plate reader. The data are expressed as the mean ± SEM from 3 independent experiments; one-way ANOVA followed by Duncan posthoc; $^{***}p<0.001$ vs. vehicle-treated cells; $^{\#}p<0.05$ and $^{\#\#}p<0.01$ vs. H_2O_2-treated cells. C - control; CeO – polyacrylic acid-conjugated cerium oxide nanoparticles; Gd-CeO – polyacrylic acid-conjugated cerium oxide nanoparticles; H – H_2O_2; N – NAC; V – vehicle.

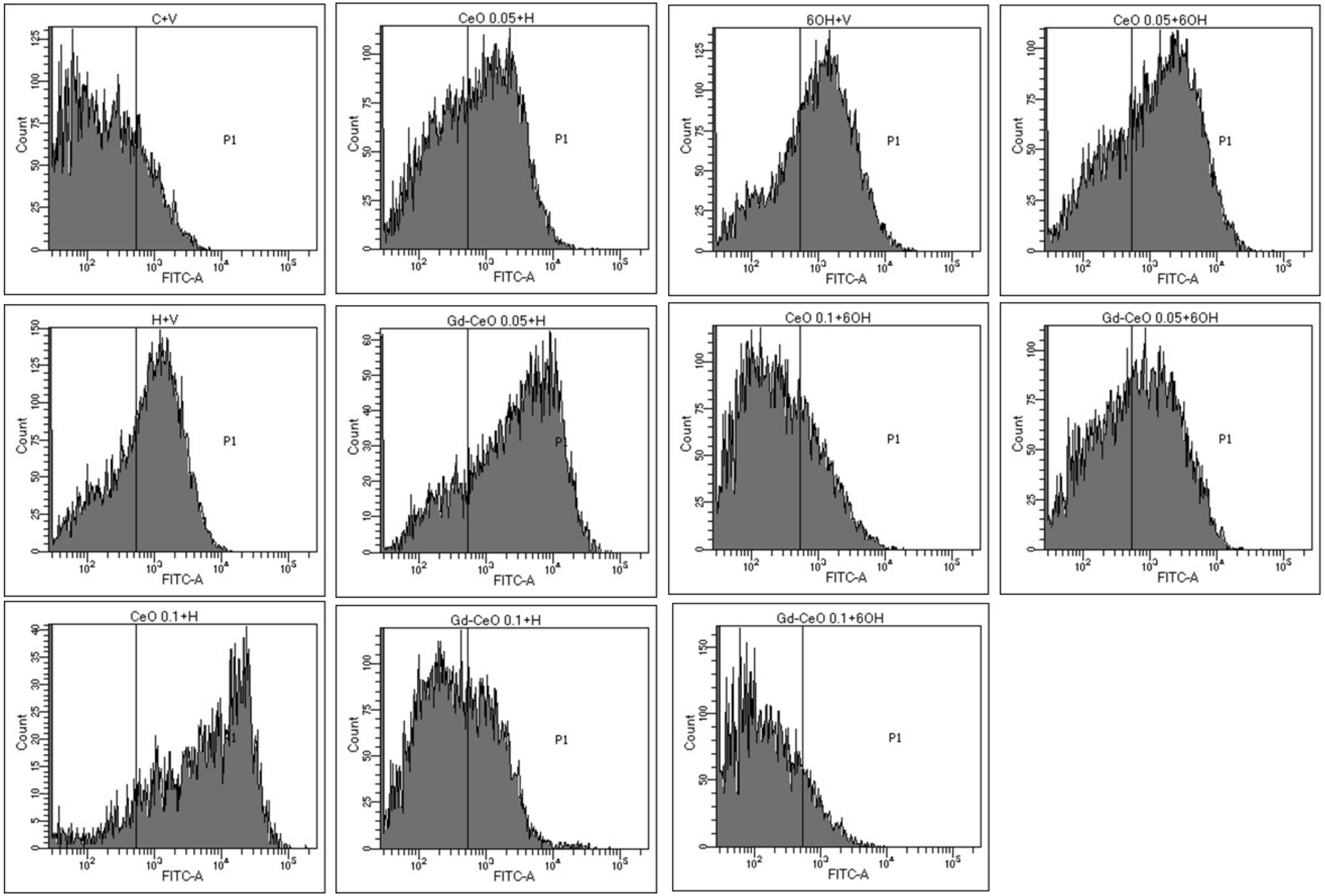

Fig. S10. Representative flow cytometry histograms of TUNEL labelling (FITC channel) for RA-SH-SY5Y cells pretreated for 30 min with vehicle (10% v/v distilled water), 0.05M and 0.1M CeO and 20% Gd-CeO NPs at dilution 10x followed by 24 h treatment with H_2O_2 (0.5 mM) or 6-OHDA (0.2 mM). 6-OH – 6-OHDA; C – control; CeO – polyacrylic acid-conjugated cerium oxide nanoparticles; Gd-CeO – polyacrylic acid-conjugated cerium oxide nanoparticles; H – H_2O_2; NPs – nanoparticles; V – vehicle.

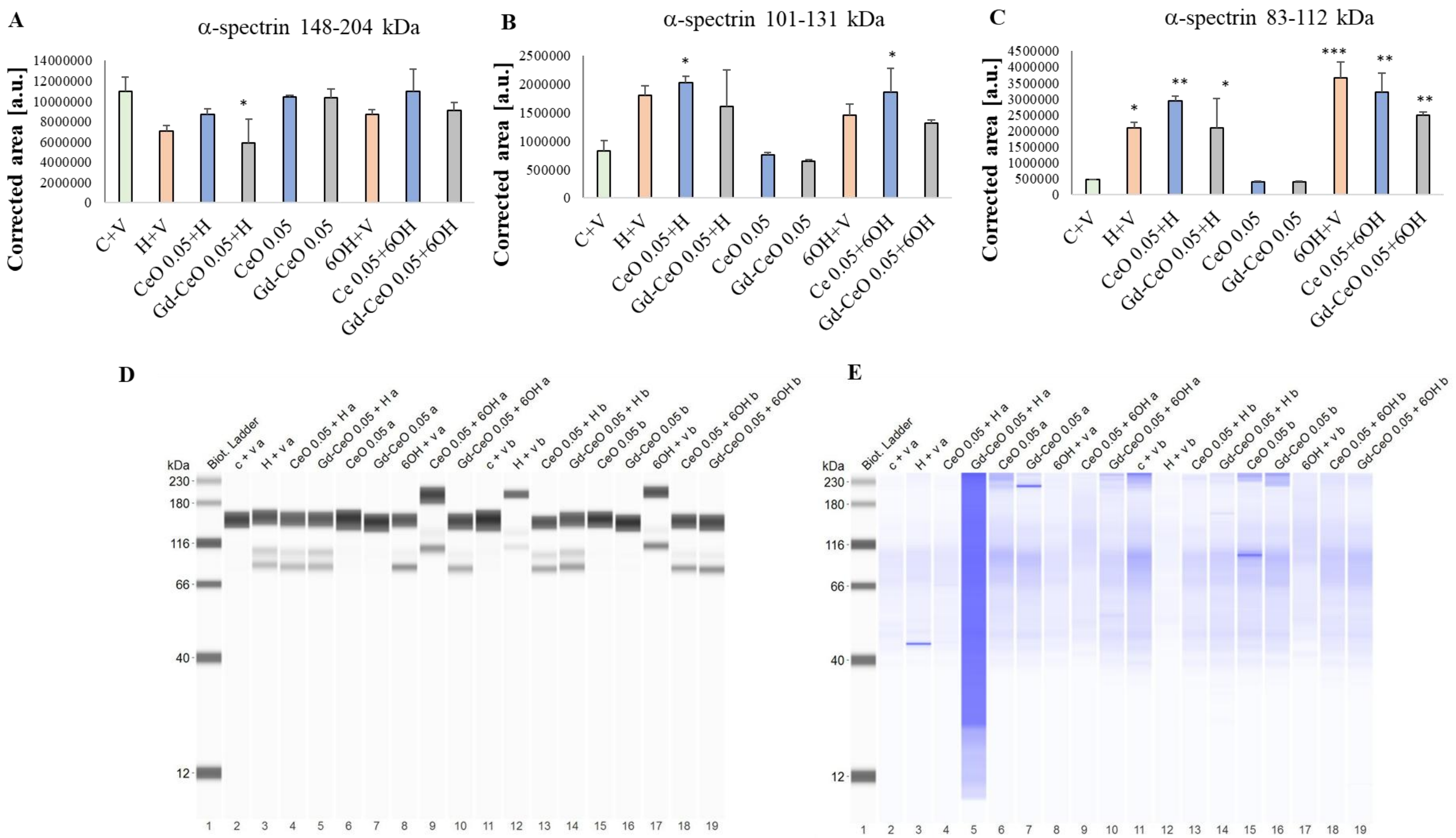

Fig. S11. The effect of CeO and 20% Gd-CeO NPs on the H_2O_2- or 6-OHDA-induced increase in spectrin α II cleavage products induced by calpains (101-131 kDa) or caspases (83-112 kDa) in RA-SH-SY5Y cells. The cells were pre-treated for 30 min. with vehicle (10% v/v distilled water), 0.05M CeO and 0.05M 20% Gd-CeO NPs at dilutions 10x followed by 18 h exposure to H_2O_2 (0.5 mM) or 6-OHDA (0.2 mM). The protein expression of total (148-204 kDa) spectrin α II and its cleavage products induced by calpains (101-131 kDa) or caspases(81-112 kDa) was measured by quantitative JESS capillary-based western blotting system (Protein Simple Jess, Biotechne). **(A-C)** Histograms of total (A), calpain (B) and caspase (C) related spectrin α II protein level; the mean corrected area ± SEM from one experiment in duplicates; one-way ANOVA followed by Duncan posthoc; $^{*}p<0.05$, $^{**}p<0.01$ and $^{***}p<0.001$ vs. vehicle-treated cells. **(D-E)** Portrayal of a traditional blot-like image with a lane view of the samples (in duplicates a, b) probed with anti-α-spectrin antibody (D) and the total protein (E). 6-OH – 6-OHDA; C – control; CeO – polyacrylic acid-conjugated cerium oxide nanoparticles; Gd-CeO – polyacrylic acid-conjugated cerium oxide nanoparticles; H – H_2O_2; NPs – nanoparticles; V – vehicle
